# Mesoporous Bioactive Glasses in Cancer Diagnosis and Therapy: Stimuli‐Responsive, Toxicity, Immunogenicity, and Clinical Translation

**DOI:** 10.1002/advs.202102678

**Published:** 2021-11-19

**Authors:** Esmaeel Sharifi, Ashkan Bigham, Satar Yousefiasl, Maria Trovato, Matineh Ghomi, Yasaman Esmaeili, Pouria Samadi, Ali Zarrabi, Milad Ashrafizadeh, Shokrollah Sharifi, Rossella Sartorius, Farnaz Dabbagh Moghaddam, Aziz Maleki, Hao Song, Tarun Agarwal, Tapas Kumar Maiti, Nasser Nikfarjam, Colin Burvill, Virgilio Mattoli, Maria Grazia Raucci, Kai Zheng, Aldo R. Boccaccini, Luigi Ambrosio, Pooyan Makvandi

**Affiliations:** ^1^ Department of Tissue Engineering and Biomaterials School of Advanced Medical Sciences and Technologies Hamadan University of Medical Sciences Hamadan 6517838736 Iran; ^2^ Institute of Polymers Composites and Biomaterials National Research Council (IPCB‐CNR) Naples 80125 Italy; ^3^ School of Dentistry Hamadan University of Medical Sciences Hamadan 6517838736 Iran; ^4^ Institute of Biochemistry and Cell Biology (IBBC) National Research Council (CNR) Naples 80131 Italy; ^5^ Chemistry Department Faculty of Science Shahid Chamran University of Ahvaz Ahvaz 61537‐53843 Iran; ^6^ School of Chemistry Damghan University Damghan 36716‐41167 Iran; ^7^ Biosensor Research Center School of Advanced Technologies in Medicine Isfahan University of Medical Sciences Isfahan 8174673461 Iran; ^8^ Research Center for Molecular Medicine Hamadan University of Medical Sciences Hamadan 6517838736 Iran; ^9^ Sabanci University Nanotechnology Research and Application Center (SUNUM) Tuzla Istanbul 34956 Turkey; ^10^ Department of Biomedical Engineering Faculty of Engineering and Natural Sciences Istinye University Sariyer Istanbul 34396 Turkey; ^11^ Faculty of Engineering and Natural Sciences Sabanci University Orta Mahalle, Üniversite Caddesi No. 27, Orhanlı Tuzla Istanbul 34956 Turkey; ^12^ Department of Mechanical Engineering University of Melbourne Melbourne 3010 Australia; ^13^ Department of Biology Science and Research Branch Islamic Azad University Tehran 1477893855 Iran; ^14^ Department of Pharmaceutical Nanotechnology School of Pharmacy Zanjan University of Medical Sciences Zanjan 45139‐56184 Iran; ^15^ Australian Institute for Bioengineering and Nanotechnology The University of Queensland Brisbane 4072 Australia; ^16^ Department of Biotechnology Indian Institute of Technology Kharagpur 721302 India; ^17^ Department of Chemistry Institute for Advanced Studies in Basic Sciences (IASBS) Zanjan 45137‐66731 Iran; ^18^ Istituto Italiano di Tecnologia Centre for Materials Interface Pontedera Pisa 56025 Italy; ^19^ Institute of Biomaterials University of Erlangen‐Nuremberg Erlangen 91058 Germany; ^20^ Chemistry Department Faculty of Science Shahid Chamran University of Ahvaz Ahvaz 6153753843 Iran

**Keywords:** cancer therapy, diagnosis, gene and drug delivery, immunotherapy, mesoporous bioactive glasses, toxicological profile

## Abstract

Cancer is one of the top life‐threatening dangers to the human survival, accounting for over 10 million deaths per year. Bioactive glasses have developed dramatically since their discovery 50 years ago, with applications that include therapeutics as well as diagnostics. A new system within the bioactive glass family, mesoporous bioactive glasses (MBGs), has evolved into a multifunctional platform, thanks to MBGs easy‐to‐functionalize nature and tailorable textural properties—surface area, pore size, and pore volume. Although MBGs have yet to meet their potential in tumor treatment and imaging in practice, recently research has shed light on the distinguished MBGs capabilities as promising theranostic systems for cancer imaging and therapy. This review presents research progress in the field of MBG applications in cancer diagnosis and therapy, including synthesis of MBGs, mechanistic overview of MBGs application in tumor diagnosis and drug monitoring, applications of MBGs in cancer therapy ( particularly, targeted delivery and stimuli‐responsive nanoplatforms), and immunological profile of MBG‐based nanodevices in reference to the development of novel cancer therapeutics.

## Graphical abstract

Cancer targeted therapy for osteosarcoma, utilizes multifunctional mesoporous bioactive glasses (MBG) theranostic systems. It includes 1) administration of MBG through the artery as a multifunctional drug delivery system, 2) tumor targeted delivery and stimuli‐responsive MBG for cancer therapy, 3) tumor diagnosis and drug monitoring, and 4) induction of apoptosis to promote apoptotic cell death and induction of bone regeneration.

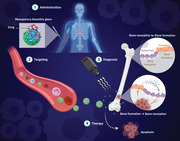

## 1 Introduction

As the second leading cause of death worldwide, cancer is a complex disease group that is still largely incurable.^[^ ^1^ ^]^ Multiple strategies, including surgery, chemotherapy, radiotherapy, hormone therapy, and targeted therapies, have been developed due to high mortality and morbidity resulting from cancer.^[^ ^2^ ^]^ Surgery is a mostly used approach for initial stages when cancer cells have not diffused to distant cells and tissues. However, in the later stages of cancer, surgery is not often recommended due to its invasive nature.^[^ ^3^, ^4^, ^5^ ^]^ Chemotherapy uses chemicals to kill or block the growth of undetectable cancer microenvironment and free cancer cells and restrict their migration. As cancer cells grow and progress faster than the healthy ones, they are the most likely targets to be affected by chemotherapeutics. However, chemotherapeutics does not differentiate between cancer and the healthy cell types.^[^ ^6^ ^]^ These unwanted side effects call upon the need for novel methods to increase the potential of chemotherapy, such as using controlled delivery systems or combination therapy with other antitumor agents.^[^ ^7^, ^8^ ^]^ Moreover, for those patients who cannot benefit from surgery or radiotherapy, hormone therapy provides an efficient treatment to slow or stop the growth of hormone‐dependent cancers. However, as hormone therapy changes the levels and function of hormones, it can lead to unwanted side effects and organ dysfunction.^[^ ^9^ ^]^

Although conventional therapies have provided considerable benefit for removing primary tumors, incidence of cancer relapse are still a frequently encountered that result from remaining malignant cells and the presence of cancer stem cells. Therefore, alternative treatment strategies warrant eradication of the resistant cancer cells.^[^ ^10^ ^]^ Nanotechnology has established a cutting‐edge framework for developing unique materials with tailored characteristics and translational potentials in cancer diagnosis, screening, and treatment. Nanotechnology, primarily based on nanoparticles, has a role in innovative cancer treatment methodologies such as targeting delivery, photothermal therapy, and photodynamic therapy.^[^ ^11^, ^12^, ^13^, ^14^, ^15^ ^]^ There is a growing interest in the development of targeted therapies for cancer. These promising strategies employ drugs and active biomolecules to identify and target tumor cells more precisely and effectively.^[^ ^16^ ^]^ Concerning most solid cancers, photodynamic therapy (PDT) has emerged as a possible therapeutic alternative owing to its high specificity and low risk of adverse effects compared with conventional therapies. Photothermal therapy (PTT), fundamentally an extension of photodynamic therapy, is a noninvasive treatment approach with high selectivity toward solid tumors.^[^ ^17^, ^18^ ^]^

In recent decades, bioactive glasses (BGs) have been introduced as multifunctional systems for various applications, including soft and hard tissue engineering, angiogenesis, orthopedic implant coatings, and drug/growth factor delivery.^[^ ^19^ ^]^ BGs are synthetic biomaterials with exceptional characteristics such as biocompatibility, controllable degradation rate, osteoconductivity, besides exhibiting antibacterial and angiogenic attributes. Furthermore, they can be reinforced with various biologically active elements, such as magnesium, zinc, strontium, and fluorine, allowing a broad scope of functions and applications.^[^ ^20^ ^]^

Mesoporous bioactive glasses (MBGs), as a special class of BGs, have attracted considerable attention to target cancer cells with excellent stability and high drug loading capacity presenting a controlled drug release system into the malignant tumor.^[^ ^21^ ^]^ MBGs possess a similar structural and textural characteristics compared with silicate‐based mesoporous materials besides sharing similar composition as conventional bioglass.^[^ ^22^ ^]^ Their capability remains limited to the delivery of antitumor, anti‐inflammatory, antibacterial drugs, and other therapeutics such as genes, peptides, and growth factors.^[^ ^22^, ^23^, ^24^ ^]^ Facile synthesis techniques have been developed to provide MBGs with controllable drug delivery capability, high drug encapsulation efficiency, and drug loading capacity. Ion released from MBG have been also shown to contribute to specific therapeutic outcomes, both in tissue engineering^[^ ^25^ ^]^ and cancer therapy.^[^ ^23^ ^]^ Cost‐effectiveness, greater sustainability of MBGs, and their controllable features make them potential systems for antibacterial, tissue engineering, and cancer drug delivery applications.^[^ ^26^, ^27^ ^]^ Moreover, MBGs enable the simultaneous delivery of therapeutic ions and drugs delivery to synergistic outcomes.^[^ ^28^ ^]^

The promising applications of MBGs as easy‐to‐use vehicles for the targeted delivery of desired therapeutic and diagnostic cargos have been documented in a limited number of studies.^[^ ^29^, ^30^, ^31^, ^32^, ^33^, ^34^, ^35^, ^36^ ^]^ Such emerging encouraging results indicate that these nanoscale materials should widely be deployed soon to realize their potentials. Due to the unique physicochemical properties of MBGs, a considerable number of multifunctional nanoplatforms for theranostics (the combination of a diagnostic signal with a therapeutic effect) can be designed and developed.^[^ ^23^ ^]^ In this regard, their well‐defined mesoporous structure with adjustable porosity, pore‐volume, surface area, size, shape, as well as their easy synthesis and surface modification, make MBGs ideal candidates in targeted cancer therapies.^[^ ^37^ ^]^

**Figure 1:**
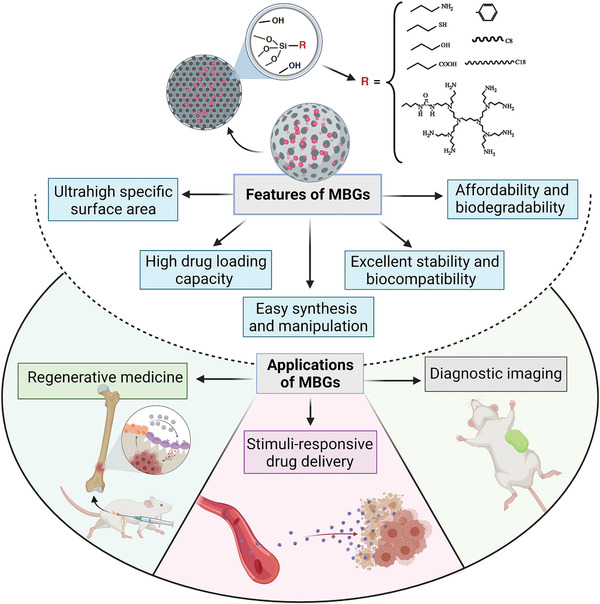
An overview of mesoporous bioactive glass (MBG) properties and their potential biomedical applications.

This review covers the recent advancements, challenges and prospects of different strategies for the synthesis of MBG nanostructures and their applications in tissue regeneration and drug delivery for cancer treatment (**Figure** **1**). Although the applications of bioceramics in cancer therapy have already been proposed for over a decade,^[^ ^38^, ^39^ ^]^ there have been tremendous advances in the past few years, particularly in the development of MBGs as a potential next generation biomaterial. In this regard, the main focus of this article is on stimuli‐responsive systems based on MBGs with diagnostic imaging and/or therapeutic purposes that have been conjugated with various biomolecules such as antibodies, peptides, and aptamers to improve specific delivery to tumors or tissues. These strategies along with the broad range of advantages of MBGs such as versatility, nontoxicity, biocompatibility, and biodegradability, aimed to reduce side effects and enhance therapeutic efficacy through more precise cancer treatments. Moreover, the immunogenicity and potential toxicity of MBGs are outlined. We have then provided insight into challenges and perspectives in the field of MBGs to highlight current and future research areas with relevance for paving the way for clinical translation.

## 2 The Significance of MBGs: Overview on the Role of Cancer Treatment

In recent years, considerable research efforts have been directed to develop nanocomposites based on BGs with enhanced therapeutic agent delivery.^[^ ^40^, ^41^, ^42^ ^]^ MBGs with highly ordered pore structures have been prepared in various shapes, from discs, fibers, and spheres to composites and scaffolds.^[^ ^22^, ^43^ ^]^ Compared to nonporous BG materials, MBGs have a higher surface area and pore volume, allowing them to carry larger quantities of drug molecules and to enable more sustained release durations.^[^ ^44^ ^]^ Synthesis parameters, including temperature, reaction time, pH, and concentration of template, are crucial to tune the mesostructures of MBGs.^[^ ^45^ ^]^ These MBG characteristics ultimately allow for the highly efficient loading of diverse hydrophobic and hydrophilic agents toward well‐controlled delivery applications. Moreover, the ease of synthesis and scale up make MBG being highly desirable for industrial applications.^[^ ^46^ ^]^

The silanol groups exposed on the MBGs surface provide anchoring points for further modification with different functional groups via either noncovalent and or covalent binding. Depending on the type of functional groups applied on the MBGs surface, they can promote drug delivery capabilities and enhance cell response.^[^ ^24^, ^47^, ^48^ ^]^ The bioactivity and biocompatibility of MBGs, have been widely examined for various biomedical applications. An ideal MBG should possess excellent biocompatibility and promote cellular adhesion, dispersion, migration, and proliferation to enhance tissue regeneration.^[^ ^49^, ^50^ ^]^ Likely, MBGs must have the features of a biosafe material to ensure safe drug delivery into tumor cells with no in vitro and in vivo toxicity.^[^ ^42^, ^51^ ^]^

MBGs usually exhibit higher bioactivity than traditional sol–gel BGs with a similar composition. For instance, hydroxyapatite formation on the surface of MBG 58S is faster than on conventional sol–gel BG.^[^ ^40^, ^41^, ^42^ ^]^ Taken together, MBGs could be considered as suitable carriers for local delivery of drugs/factors due to their ability to interact with specific tissues.^[^ ^24^, ^28^, ^32^ ^]^ These unique features (e.g., biodegradability, biocompatibility, drug‐delivery, osteogenic, and angiogenic potential) make MBGs desirable candidates for biomedical applications including cancer therapy.^[^ ^41^, ^52^, ^53^ ^]^ In recent years, many preclinical studies have developed stable MBGs with controlled delivery of drugs, desirable biosafety, and efficient suppression of tumor growth to pave the way for clinical applications.^[^ ^34^, ^54^, ^55^, ^56^ ^]^

**Figure 2:**
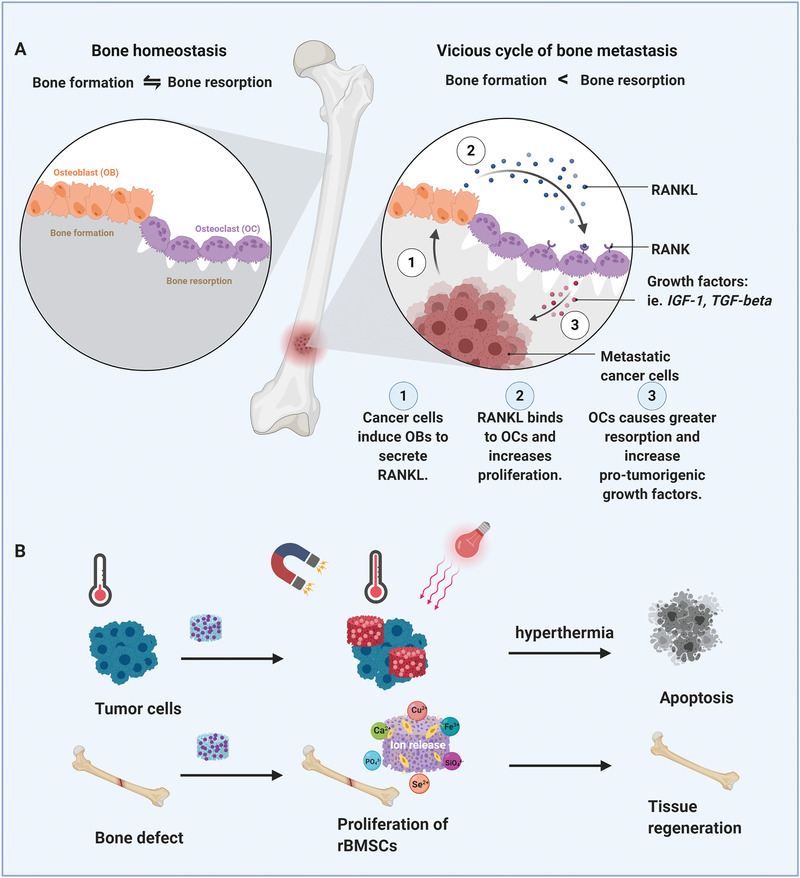
Osteoblast and osteoclast cells' activity in healthy and cancerous bone plus bone cancer treatment through magnetic and light‐responsive materials. A) The Schematic indicates the normal and abnormal functions of osteoblast and osteoclast cells in cancerous bone cells.B) The schematic illustrates simultaneous bone regeneration and cancer therapy of stimuli‐responsive MBGs.

Besides MBG applications in different cancer types, special consideration has been devoted to bone cancer rooting in the therapeutic/regenerative effects of released ions (Si, Ca, and P) from the MBGs’ structure. These ions have osteogenic and angiogenic effects leading to accelerated bone tissue regeneration.^[^ ^24^ ^]^ Bone remodeling is controlled by two forces including bone formation by osteoblast opposed to bone destruction or resorption by osteoclasts (**Figure** **2**). The intimate interaction between these cells is an essential element in bone hemostasis; for instance, if osteoclasts activity dominates, leading to decreased bone mass. Bone cancer is a complication, as it causes the osteoclast cells to proliferate and provide protumorigenic growth factors. Moreover, bone cancer can intensify osteoporosis, accelerating the deterioration of the patient's condition.^[^ ^57^ ^]^ Therefore, applying stimuli‐responsivity to MBGs is a suitable approach to deal with bone cancer. Besides bone defect regeneration, these multifunctional MBGs, like Fe‐containing MBGs, can generate adequate heat under an external magnetic field (EMF) or near‐infrared laser (NIR) to eliminate cancerous bone cells.^[^ ^58^, ^59^ ^]^

## 3 Synthesis of MBGs

**Figure 3:**
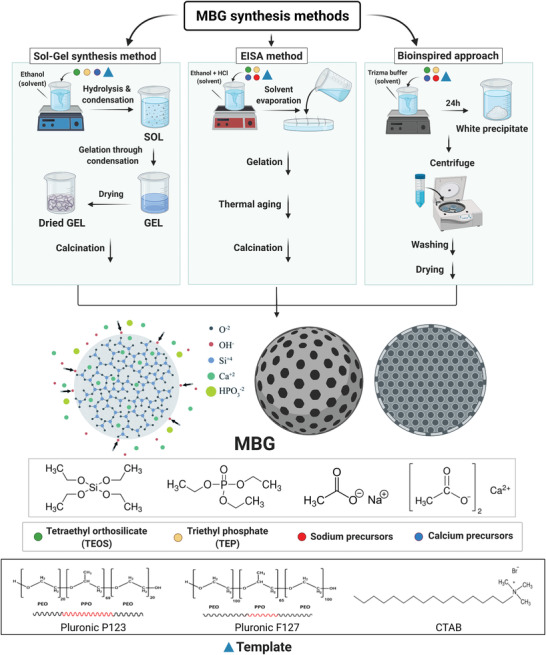
Schematic illustration of the MBGs synthesis by sol–gel process, EISA method, and bioinspired approach. CTAB: cetyltrimethylammonium bromide; HCl: hydrochloric acid; EISA: evaporation induced self‐assembly; Pluronic: an amphiphilic material based on poly(ethylene oxide)–poly(propylene oxide)–poly(ethylene oxide) (PEO–PPO–PEO) block copolymers.

Currently, there emerged three conventional methods that are widely used for the synthesis of porous BGs^[^ ^60^ ^]^ (**Figure** **3**). Sol–gel‐based strategies provide a low‐temperature production of MBGs with flexible compositions. In this way, metal–organic and metal salt precursors can be incorporated into the glass network for therapeutic applications.^[^ ^61^, ^62^, ^63^ ^]^ As an example of these metals, copper (Cu) has the potential to promote angiogenesis and osteogenesis on the one hand and add antibacterial and anti‐inflammatory properties to the glass composition on the other hand.^[^ ^64^ ^]^ Besides, Cu can also import anticancer properties.^[^ ^65^ ^]^ Although incorporating silver (Ag) into the MBGs affects morphology, specific surface area, and textural properties, this ion has anticancer activity and apoptotic effects against cancerous cells.^[^ ^66^, ^67^ ^]^ Cerium (Ce) has unique chemical, physical, and biological properties, including angiogenic, anticancer, and antibacterial.^[^ ^68^, ^69^ ^]^ Zinc (Zn) is an essential trace element for the structure and function of several macromolecules with anticancer properties which is caused by its antioxidant properties.^[^ ^70^ ^]^ As a semimetal element, incorporating gallium (Ga) into the MBG structure is a possible approach to treating certain cancers thanks to its anti‐neoplastic and anticancer activities without any significant unfavorable response.^[^ ^71^, ^72^ ^]^

Sol–gel process has shown flexibility, high yield, and affordable synthesis of well‐dispersed BGs with full control of the product's composition and structure.^[^ ^61^, ^73^, ^74^ ^]^ By employing structure‐directing agents in sol–gel processing, MBGs with controlled morphology (e.g., spheres, fibers) can be synthesized. However, particle aggregation of the resulting MBGs remains the main challenge to achieving homogenous particle dispersion.^[^ ^26^, ^75^ ^]^

Evaporation‐induced self‐assembly (EISA) method, which comprises block copolymers as nonionic structure‐directing agents, is a commonly applied strategy in sol–gel processing to construct ordered mesoporous materials.^[^ ^60^ ^]^ In this process, changing various parameters such as solvent composition, temperature, and copolymers structures and molecular weights can influence the porous structure and morphology of MBGs.^[^ ^76^ ^]^

The traditional sol–gel and EISA methods mostly involve using toxic organic solvents and a high‐temperature process to fabricate bioactive glass scaffolds with various pore architectures ranging from meso‐ to macroporous structures.^[^ ^77^, ^78^ ^]^ Conventional methods require extremes of temperature and pH levels; however, the bioinspired synthesis of porous materials is performed in an aqueous solution under ambient conditions. This method would offer researchers better control over the process of synthesis to optimize silicate‐based bioactive glass materials' morphology and chemical properties.^[^ ^21^, ^79^ ^]^ There are many advantages associated with the proposed production method of MBGs, for example: cost‐effectiveness, environmental friendliness, and energy saving.^[^ ^40^ ^]^

The importance of templates in tuning the physicochemical properties of MBGs and affecting their bioactivity is critical. Various templates in different synthesis protocols can be divided into the following types: linear polymers (polymethyl methacrylate, terylene, cellulose),^[^ ^80^, ^81^ ^]^ dendritic polymers (polyamidoamine dendrimer),^[^ ^82^ ^]^ surfactants such as cetyltrimethylammonium bromide (CTAB) and triblock copolymers like (P123, F127),^[^ ^49^, ^83^, ^84^ ^]^ sponges (polyurethane),^[^ ^85^ ^]^ emulsions,^[^ ^86^ ^]^ and DNA (calf thymus DNA)^[^ ^78^ ^]^ (Figure 3). However, ionic surfactant CTAB and nonionic surfactants made by triblock copolymers based on polyethylene oxide (PEO) and polypropylene oxide (PPO) such as P123 ((PEO)_20_–(PPO)_70_–(PEO)_20_) and F127 ((PEO)_100_–(PPO)_65_–(PEO)_100_) are the most frequently used templates due to their micellar formation ability.^[^ ^24^ ^]^

Depending on the surfactant nature, for instance, the molecular weight, and hydrophilic–lipophilic balance (HLB), as well as the surfactant concentration, mesoporous architectures in highly ordered, partially ordered or even totally disordered manner could be obtained .^[^ ^87^ ^]^ By way of example, F127 with molecular weight of 12 600 Da leads to MBGs with pore size twice larger than the ones in MBGs obtained through CTAB templating with much smaller molecular weight (364.4 Da).^[^ ^88^ ^]^ Also, the concentration of these templates could also play a pivotal role in determining the textural properties of MBGs. For example, it was observed that at 0.4 × 10^−3^ and 0.6 × 10^−3^ m CTAB and 2 g P123, respectively, BG formation is not observed due to the absence of micellization at below surfactant CMC.^[^ ^89^, ^90^ ^]^

As a result, commonly used surfactants such as CTAB, P123, and F127 in the synthesis procedure could produce various types of pore, such as worm‐like, hexagonal, and cubic pore structures.^[^ ^91^ ^]^ Moreover, using a particular template via different routes of synthesis can also resulted in MBGs of different morphology and textures.^[^ ^60^ ^]^ In this regard, using surfactants such as CTAB, F127, and P123 as structure‐directing agents for MBG synthesis, in many studies has resulted in MBGs with diverse final mesopore size, volume, and surface area, ranging from 2 to 10 nm, 0.4 to 0.7 cm^3^, and 150 to 1000 m^2^ g ^−1^, respectively.^[^ ^72^ ^]^

## 4 Cancer Diagnosis and Drug Monitoring

**Figure 4:**
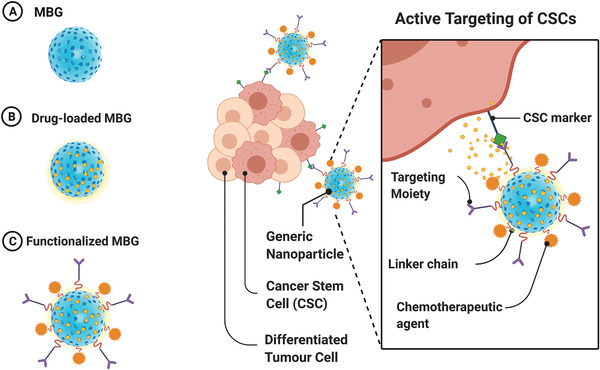
Schematic illustration of the A) MBGs, B) drug‐loaded MBGs, and C) functionalized MBGs in diagnostic and drug delivery. CSCs: cancer stem cells.

The diagnosis and prognosis of cancers, as well as their treatment options, are complex. Early detection of cancers in some cases can lead to better recovery of patients or even no need for complex treatments such as surgery, radiotherapy, chemotherapy, and immunotherapy.^[^ ^27^ ^]^ Hence, the development of new diagnostic methods (noninvasive and minimally invasive) can be substantial. In cancer diagnosis, patient pathways for presentation and initial management in the primary care unit are crucial. Delaying the patients to check for symptoms, consulting with health care professionals, or delaying diagnosis can lose the golden times of cancer therapy and may reduce the chances of rescuing patients in the early stages of the disease. Therefore, a precise and timely cancer diagnosis is a key parameter that improves the prognosis and reduces the cancer patient's psychological distress.^[^ ^92^ ^]^ In addition to the MBG nanosphere usages in the cancer diagnosis domain, their applications as modified multifunctional nanospheres in the field of controlled delivery of cancer drugs are also noteworthy (**Figure** **4**).

There is little published research on the diagnostic performance of MBGs, although their operating system is similar to that used in BG‐based biomaterials. As such, more research in this field is expected in the near future. Overall, most of the cancer diagnostic agents reported in the literature are combined with hyperthermia therapy, photothermal therapy, tissue regeneration, or remedial methods, few studies on diagnostic alone are addressed in this section.

**Figure 5:**
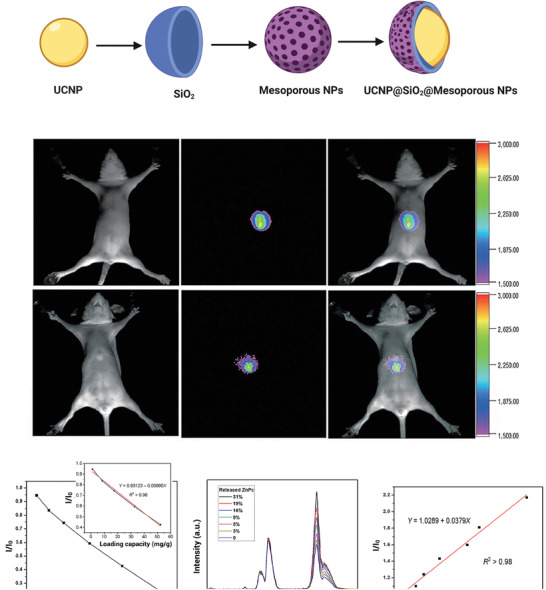
Diagnosis application of bioactive glasses. A) Schematic illustration of the structure of UCNP@SiO_2_@MBG NPs. B) The luminescence in vivo imaging of athymic nude mice with intravenous injections of UCNPs@SiO_2_@MBG. Resolution/sensitivity of in vivo imaging enhanced by incorporating Ca (top row of in vivo imaging combined with Ca). All images were obtained under the same instrumental conditions (powder density ≈ 120 mW cm^−2^ on mice's surfaces). C) The loading capacity range of UCNPs@SiO_2_@MBG/ZnPc. D) MBG/UCNP nanocomposites' intensity versus release of ZnPc. E) Linear range of *I*/*I*_o_ response for each release percentage. UCNPs: upconversion nanoparticles; ZnPc: zinc phthalocyanine. (B–E) Reproduced with permission.^[^ ^93^ ^]^ Copyright 2016, Springer Nature.

Upconversion nanoparticles (UCNPs) are a subset of nanomaterials commonly doped with lanthanide ions. Notable features of these nanoparticles include the ability to transfer two or more photons to higher energies. Due to the emission capability of upconversion nanoparticles in the visible/NIR domain, they can be applied with MBGs to monitor and deliver anticancer drugs in tumor cells. The UCNP@MBG nanocomposites showed red emission, which grants a cavernous tissue penetration for photoluminescence imaging. Zinc phthalocyanine as an anticancer drug exhibiting a broad absorption band with a maximum wavelength of 660 nm. This wavelength is conveniently relative to the emission spectrum of Er^3+^, which helps to an effective energy transfer from MBG/UCNP nanocomposites to zinc phthalocyanine. Loading of anticancer drugs affects the fluorescence intensity and fluorescence quenching of the nanocomposite occurs (**Figure** **5**).^[^ ^93^ ^]^

**Figure 6:**
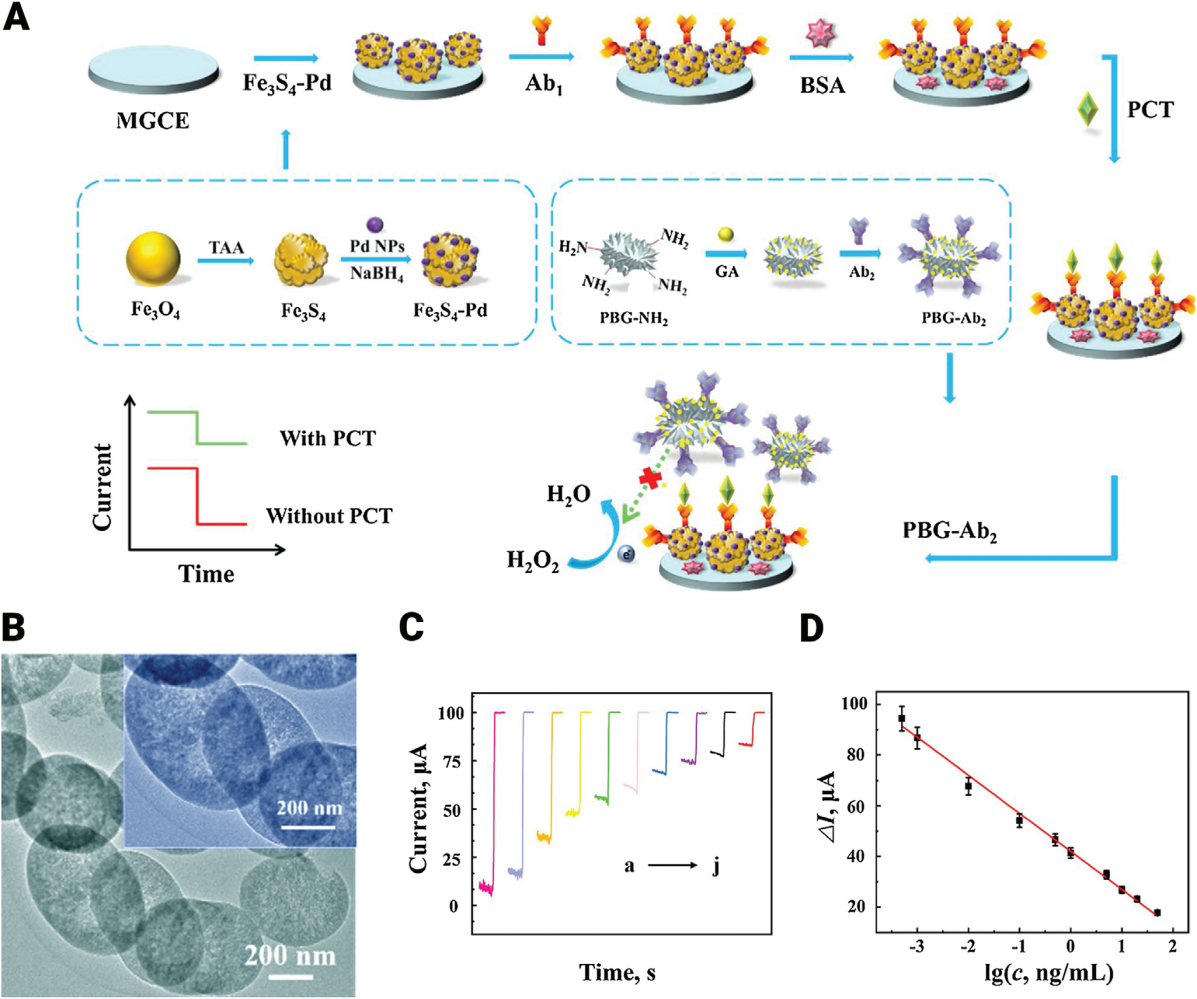
Fabrication and applications of immunosensors based on bioactive glasses. A) Schematic of experimental pathways for preparation of electrochemical immunosensor and amperometric responses. B) TEM image of slit‐shaped PBG materials. C) Curve of amperometric responses and D) calibration curve of the prepared immunosensor for PCT detection with a wide linear range (500 fg mL^−1^ to 50 ng mL^−1^, Error bars or RSD for five measurements were calculated). MGCE: magnetic glass carbon electrode; PCT: procalcitonin; GA; glutaraldehyde; Ab_1_: the primary antibodies; PBG‐Ab_2_; the secondary antibodies‐pineal mesoporous bioactive glass. Reproduced with permission.^[^ ^96^ ^]^ Copyright 2020, Elsevier.

Functionalized MBGs have been exploited for signal amplification of procalcitonin diagnosis. Procalcitonin is vital in cancer diagnosis with clinical signs of fever and elevated C‐reactive protein levels.^[^ ^94^, ^95^ ^]^ Pd@Fe_3_S_4_ with high catalytic efficiency was connected to NH_2_‐functionalized pineal MBG to enhance the detection signal of electrochemical immunosensor. Moreover, MBGs possess highly hindrance effect, which affects the sensitivity and signal responses.^[^ ^96^ ^]^ In another study, multifunctional Tb‐doped MBG nanospheres synthesized via a facile sol–gel methodology exhibited controlled doxorubicin (DOX) delivery. The drug‐releasing kinetic from Tb/MBG nanospheres can be impressively adjusted by pH changing and variation of doping‐ion concentrations. Nevertheless, Tb/MBG nanospheres possess potential for use as a compelling candidate of therapy caused by malignant cancers.^[^ ^97^ ^]^ **Figure** **6** illustrates the experimental pathways for the preparation of electrochemical immunosensor and amperometric responses. MBGs were also functionalized by folic acid and applied for targeting cancer cells. Existing of folate receptors with high profusion on cancer cells is the reason selection of these functional groups. The fluorescein isothiocyanate as a fluorescent label was covalently attached to MBG‐FA. The results illustrate that receptor‐mediated endocytosis can be induced by two cancer‐cell lines (viz., human cervix carcinoma HeLa and fibroblast cell line L929) and it enhances drug delivery efficacy.^[^ ^98^ ^]^

## 5 Cancer Therapy

### 5.1 Targeted Nanoplatforms

#### 5.1.1 Chemotherapeutic Drug Delivery

Conventional drug administration approaches (i.e., oral administration or injection) can cause fluctuation of drug concentration in blood. As a result, the drug concentrations are timely ineffective as they are lower than the therapeutic dose and causes more adverse effects when high doses are applied.^[^ ^99^ ^]^ Moreover, there are concerns about the lack of effective dose accumulation in target organs and higher dose accumulation in nontarget organs.^[^ ^100^ ^]^ Local drug delivery systems have been introduced to overcome the deficits of conventional drug delivery systems. The advantages of the local drug delivery system are high drug delivery efficiency, continuous treatment, reduced toxicity, and convenience to the patients.^[^ ^101^ ^]^

MBGs have attracted much attention in drug delivery due to their, large surface area, high pore volume, and excellent biocompatibility.^[^ ^101^, ^102^ ^]^ In addition to introducing angiogenesis and osteoblastic factors by MBG nanocomposites, therapeutic drugs for cancer treatment could also be easily introduced into the site of disease.^[^ ^103^ ^]^ MBGs can efficiently host drugs through 1) adsorption on the external surface, 2a) entrapment inside the mesoporous structure via noncovalent binding, or 2b) covalent bonding, and 3) drug lying at the window of the MBGs.^[^ ^104^, ^105^, ^106^ ^]^ For example, MBGs can adsorb drugs into the pores or their external surfaces through chemical bonding via the electrostatic attraction of Si—OH and P—OH groups on the MBG surface and the amino groups of the therapeutic molecules.^[^ ^107^ ^]^

Chemotherapy is a conventional and frequently used method for cancer therapy. Chemotherapy operates with different mechanisms, but the primary performance is to destroy growing cells (i.e., in most cases both tumor and normal cells), leading to side effects including bone marrow suppression, hair loss, and gastrointestinal reactions.^[^ ^108^ ^]^ Targeted platforms generally include two approaches to reach the intended site. Passive targeting is based on the circulation of nanoparticles in the blood for a long time, avoiding renal clearance, and selective accumulation in tumor tissues due to leaky tumor blood vessels and poor lymphatic drainage. Active targeting uses functionalized nanoparticles using active ligands such as proteins, peptides, and antibodies to allow nanoparticles to precisely reach the tumor site.^[^ ^109^ ^]^

As the idea of using MBGs as a drug delivery system has been introduced, the concept of using them as an anticancer drug delivery system was raised. MBGs have a great potential in bone tissue engineering; hence they can be beneficial in bone tumor therapy and bone defect regeneration simultaneously.^[^ ^31^ ^]^

Although these MBGs have several unique features and have been successfully employed for diverse biomedical applications, controversy still exists regarding their effectiveness as anticancer drug delivery systems.^[^ ^25^ ^]^ Many recent studies have been conducted to assess the ability of MBGs in anticancer drug delivery. They are divided into two main categories. The first group focuses on the drugs and MBG interactions (i.e., drug loading and releasing kinetics and properties) summarized in **Table** **1**; and, the second group has progressed further and evaluated drug‐loaded MBGs interplay with different cancerous cell lineages (**Table** **2**).

**Table 1:** The MBGs delivery systems for cancer drug delivery applications

Bioglass/MBGs	Apatite formation potential	Biocompatibility	Drug	Drug loading properties	Drug release properties	Outcome	Refs.
MBG	MBG nanospheres enhanced HA mineralization potential	Cytotoxicity assessed to be minimal on MC3T3 cells with all concentrations of MBG nanospheres (0.1, 0.2, or 0.5 mg mL^−1^)	DOX	Drug encapsulation efficacy of 63.6% was measured; As the surface area and pore volume increases, drug loading potential increases	Drug release has an initial burst pattern followed by a sustained release Drug release kinetics is in contrast with release environments pH	MBG can be used as a superior delivery system for cancer therapy and simultaneous bone tissue regeneration	[110]
Terbium (Tb)/MBG	Tb/MBG induces HA‐mineralization in SBF	MBG showed no toxic effect on MC3T3 cells, 0.5Tb/MBG showed no significant inhibition at low concentrations (50 µg mL^−1^ and 100 µg mL^−1^), 1 Tb/MBG showed significant inhibition on cell viability at 200 µg mL^−1^ concentration	DOX	Tb/MBG nanospheres exhibit higher specific area and pore volume that facilitate drug loading into nanospheres inner mesopores	Incorporated Tb into MBG causes a significant decrease in DOX release rate in different release solution pH, and DOX accumulative release of Tb/MBG was lower than MBG Drug release rate of all samples increased with a decrease in pH values.	Tb/MBG has the potential to be used in bone tissue regeneration, bone tumor treatment, and bone defect repair	[97]
Sm(Samarium)/MBG/Alginate	Apatite formed on the Sm/MBG/alginate surface	Not reported (N.R.)	DOX	0Sm/MBG/Alginate, 0.5Sm/MBG/Alginate and 1Sm/MBG/Alginate showed loading efficacy of 44.8%, 56.7%, and 41.4% Respectively	The drug release kinetic of Sm/MBG/Alginate increased in higher pH values 1Sm/MBG/Alginate showed the highest release rate followed by 0Sm/MBG/Alginate and 0.5Sm/MBG/alginate	Sm/MBG/Alginate can act as a smart delivery system that changes released drug concentration in a pH‐dependent manner.	[111]
Selenium/MBG	Selenium can enhance the ability to form HA on Se/MBG surface	N.R.	DOX	5Se/MBG loading efficiency (50%) was higher than 0Se/MBG (38.8%), while pore sizes were similar	All samples showed an initial burst release followed by a sustained release, and 5Se/MBG drug release kinetics was significantly lower than 0Se/MBG during 72 h	Se/MBG particles showed high drug loading efficacy and controllable drug release profile for bone cancer therapy	[112]
CuO/MgO MBG–Zn	HA formed on all MBG samples surface	Samples with higher magnesium content showed less toxicity; furthermore, at low concentrations (7.8125 µg mL^−1^), all samples showed no toxicity toward the osteosarcoma cell line (MG63)	DOX	DOX loading was in contrast with MBGs magnesium content and drugs concentration; at 100 µg mL^−1^ concentration, DOX was not loaded in all MBG samples	N.R.	MBGs can be used as a drug delivery system owing to their suitable properties	[113]
CuO/ZnO/MBG	Apatite layer formation on surface observed in All MBGs with different Cu/Zn concentrations	N.R.	DOX	DOX loading was commensurate with the copper content DOX loading was in contrast with zinc content and drug concentration (between evaluated drug loading concentrations; 20, 40, 60, and 80 µg mL^−1^)	N.R.	MBGs can be used as a drug delivery agent	[114]
MBG/polyurethane	N.R.	All composites did not show significant viability reduction on normal human fibroblast (NHFB) cells	Imatinib	N.R.	All different MBG/polyurethane nanocomposites exhibited extended drug release (52–84%) being in contact with physiological fluid for three weeks	MBG/polyurethane nanocomposites with various compositions can be used for drugs long‐term sustained release	[36]

N.R.: not reported; Sm: samarium; DOX: doxorubicin; Tb: terbium; HA: hydroxyapatite.

**Table 2:** MBG‐based delivery systems for cancer treatment

Bioglass/MBGs	Cancer cell type	Drug	Bone‐conduction capacity	Biocompatibility	Drug loading properties	Drug release properties	Drug delivery effect on cancer cell lineage	Conclusion	Refs.
MBG	Osteosarcoma cell line (MG63)	Imatinib (IMT)	Considerable hydroxycarbonate apatite formation and bioactivity	Not reported (N.R.)	Drug loading amount and efficiency increased with drug loading concentrations increase (from 0.2 mg mL^−1^ to 1.0 mg mL^−1^), with maximum of 77.59% for 1.0 mg mL^−1^ concentration	Drug release rate and cumulative drug release are in contrast with pH values Drug loading concentration influences the drug release profile	IMT‐MBG showed a significant inhibitory effect on MG63 cell lineage compared to MBG	IMT‐MBG has the potential for bone tissue regeneration and bone cancer treatment	[115]
MBG	Metastatic breast cancer cell line MDA‐MB‐231	Silibinin	N.R.	Relatively low cytotoxicity effect on Noncancerous breast endothelial cell line (MCF‐10A)	Optimal drug loading efficiency (61%) was obtained at 40 µg mL^−1^ silibinin concentration	Silibinin release has a burst at first hours (cumulative 16% release of the loaded drug in the initial 5 h), which continues with sustained drug release	MBG nanoparticles with silibinin can induce cytotoxicity and cause growth inhibition in breast cancer cell line MDA‐MB‐231	MBG nanoparticles loaded with silibinin has a high potential for clinical application	[34]
MBG	MG‐63 osteoblast‐like cell's	Alendronate (AL)	HA formation on the surface detected, MBG promoted ECM mineralization	MBG showed no toxicity to MG‐63 osteoblast‐like cells before loading AL	The optimal loading efficiency of 60% was obtained	AL drug delivery rate of MBG can be adjusted by MBG particles pore size	AL release from MBG potentially inhibited MG63 cell line proliferation, even at lower concentrations	MBG–AL demonstrated dual efficacy in bone regeneration and anticancerous drug delivery	[116]
MBG nanospheres	Osteosarcoma cell line (MG63)	Alendronate (AL)	MBG nanospheres and AL‐MBG promote mineralization in SBF	N.R.	MBG was able to load AL up to 17% wt. in optimal drug concentration of 1 mg mL^−1^	N.R.	Alendronate‐loaded MBG was effective in decreasing tumor cell viability even at lower alendronate concentration	MBG is a promising tool for bone regeneration and osteosarcoma treatment	[117]
Ag_2_O‐MBG	Osteosarcoma cell line (MG63)	DOX	Considerable apatite formation	Normal human fibroblast cell line in vitro biocompatibility in contrast with Ag_2_O‐MBG concentration (I IC_50_:178 µg mL^−1^)	Drug loading amount and efficiency increased with drug loading concentrations increase (from 0.2 mg mL^−1^ to 1.0 mg mL^−1^), with maximum of 83.5% for 1.0 mg mL^−1^ concentration increase and decrease in release media pH	Drug release rate and cumulative drug release amount increases with loading concentration	DOX‐Ag_2_O‐MBG significantly inhibited MG63 osteosarcoma cells viability	Ag_2_O‐MBG Nanoparticles are efficient for bone tissue regeneration and drug delivery	[118]
Fe_3_O_4_–MBG	Osteosarcoma cell line (MG63)	Mitomycin C (Mc)	Hydroxycarbonate apatite (HCA) formation	No significant cytotoxicity on normal human fibroblast (NHFB) cells at any concentration	The optimum drug loading efficiency of 93% was measured	Fe_3_O_4_–MBG cumulative release was in contrast with pH values	Mc–Fe_3_O_4_–MBG has a significant inhibitory effect on MG63 osteosarcoma cell line viability in a dose‐dependent manner (IC_50_: 12.19 µg mL^−1^)	Fe_3_O_4_–MBG is a nontoxic, biocompatible biomaterial with potential for bone tissue regeneration and drug delivery	[119]
Selenium–MBG	Osteosarcoma cell line (MG63)	DOX	Se^4+^ improves HA‐mineralization ability of Se/MBG	5Se/MBG at concentrations higher than 20 µg mL^−1^ showed significant toxicity to MC3T3‐E1 preosteoblast cells at 48 h Se/MBG and 3Se/MBG showed no toxicity toward MC3T3‐E1 preosteoblast cells	Se doping enhances the specific surface area and nanospheres pore volume; thus, 5Se/MBG and 3Se/MBG showed a higher drug loading rate	DOX release adjusted by pH and Se concentrations; lower pH values of release environment cause higher drug release rate, and Doping Se ions decrease DOX release rate	Se/MBG at different concentrations induces apoptosis in osteosarcoma cells (MG63); furthermore, Se and DOX codoped MBG nanospheres exhibit a long‐term inhibition on the viability of osteosarcoma cells (MG63)	Se/MBG has the potential in diagnostics, therapy, and clinical application owing to its tunable intrinsic toxicity, high surface area, and adjustable surface chemistry	[120]
Europium(Eu)/MBG	Osteosarcoma cell line (MG63)	DOX	Apatite formed; Eu changed the morphology of formed apatite from sheet to rod in a dose‐dependent manner	Eu/MBG enhanced viability of osteosarcoma MG 63 cells	DOX loading is dependent on specific surface area and pore size of MBG/Eu	DOX release increases with a decrease in pH Proper Eu content improves DOX release behavior besides its loading properties	Eu/MBG‐DOX shows controlled release of DOX, which inhibits MG 63 cells in long term	Eu/MBGs are a prospective candidate owing to their mesoporous structure, unique apatite formation, and controlled and adjustable drug delivery properties	[121]
Aminated MBG (AMBG)	MG‐63 osteoblast‐like cell's	Alendronate (AL)	AMBG promoted ECM mineralization	AMBG showed no toxicity to MG‐63 osteoblast‐like cells before loading AL	The optimal loading efficiency of 63% was obtained	AMBG Drug release profile was more controlled and sustained comparing MBG; furthermore, reducing mesopore size and creating attachment sites on AMBG causes a more sustained drug release	AL release from AMBG potentially inhibited MG63 cell line proliferation, even at lower concentrations	AMBG‐AL showed dual efficacy in bone regeneration and anticancerous drug delivery	[116]
Rice husk MBG (rMBG)	HeLa cancer cells	Camptothecin (CPT)	N.R.	rMBG has No toxicity up to a dose of 200 µg mL^−1^ after 24 h on normal fibroblasts (L929)	rMBG has a higher CPT loading capacity compare with MBG due to its higher pore volume CPT loading capacity of rMBG was measured 13.8% in PBS (pH 7.4) at 37 °C	A rapid drug release within the first week followed by sustained release after day 7	rMBG/CPT was cytotoxic to HeLa cancer cells after incubation for 3 h	rMBG can be used as a drug delivery vehicle, which increases CPT solubility as a hydrophobic anticancer drug	[41]
Dendritic MBG	Tumor (HepG2) cells	DOX	N.R.	Minimal damage to human normal (LO2) in vitro MBG–DOX has the potential to reduce cardiac and systemic toxicity caused by free DOX in vivo	Increase in DOX to MBG ratio increases loading amount while decreases loading efficiency	DOX and Ca^2+^ release was dependent on pH of release solution; release increases with pH decrease	Dendritic MBG have controlled drug delivery potential and shows a synergism effect with loaded DOX in tumor growth inhibition Tumor volume significantly decreased following injection of dendritic MBG nanospheres to animal model (mice) in vivo	Dendritic MBG nanospheres potentially can be used as a superior delivery system for cancer treatment	[122]
Fluorescent MBG nanoparticles (fBGn)	HeLa cancer cells	DOX	N.R.	fBGn showed no significant toxicity to HeLa cells up to 320 µg mL^−1^ fBGn in a varying dosage of (0, 5, 10, and 20 mg kg^−1^) were IV injected to nude mice, and the result showed high histocompatibility to almost all organs with no significant difference between doses and saline control group	Drug loading increased with drug concentration increase with the optimum of ≈92% Ca^2+^ ions improved loading efficiency	Drug release rate increases in an acidic environment, and Ca^2+^ enhances sustained drug release properties	fBGn‐DOX drug release has the potential to destroy the HeLa cancer cells	Drug release based on fBGn can be used for future cancer drug delivery	[123]

IMT: imatinib; N.R; not reported; IC_50_: half maximal inhibitory concentration; DOX: doxorubicin; Mc: mitomycin C; HCA: hydroxycarbonate apatite; AL: alendronate; Eu: europium; rMBG: rice husk MBG; CPT: camptothecin.

There are different critical aspects to consider MBGs as drug delivery systems. Drug loading conditions and drug release conditions are significant performance measures in relation to MBGs drug delivery characteristics assessment.

The drug uptake ability of MBGs can be manipulated in different ways. Higher loading concentrations usually lead to enhanced drug loading capacity; ^[^ ^122^, ^123^ ^]^ By contrast, some studies indicated that DOX loading capacity of MBGs decreased when the loading concentration increased.^[^ ^113^, ^114^ ^]^ The drug loading amount depends on the pore size and surface area of MBGs.^[^ ^110^, ^120^, ^121^ ^]^ The effect of therapeutic ions incorporated in MBGs on the loading capacity has been investigated. Studies concluded that selenium (Se) and copper (Cu) increased the drug loading capacity of MBGs.^[^ ^112^, ^114^, ^123^ ^]^ On the other hand, it has been concluded that higher amounts of folic acid, Mg, and Zn decrease the loading capacity of MBGs.^[^ ^41^, ^113^, ^114^ ^]^ Hence, an optimized drug loading content and efficiency can be obtained by tuning the chemical composition of the MBG carrier and tailoring structural characteristics.

**Figure 7:**
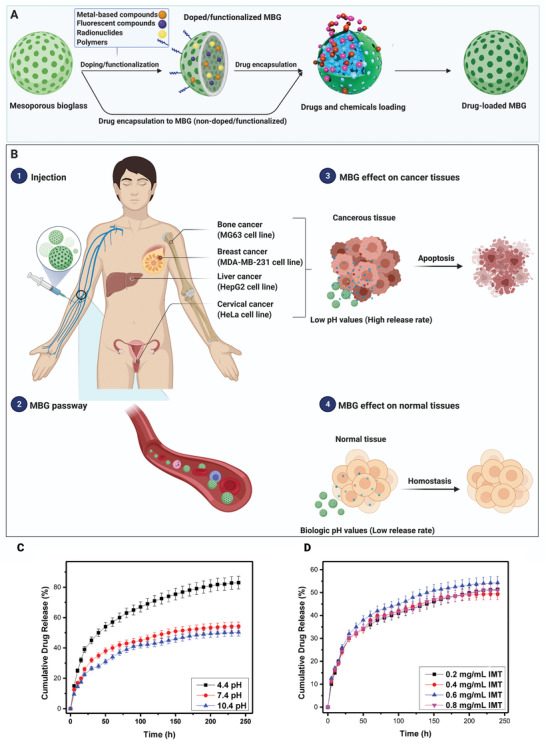
A prospective overview of MBGs application as drug delivery platform. A) Preparation of drug‐loaded MBG. B) Drug‐loaded MBGs affect normal cells and various cancer cell lines. C) Drug release properties against different pH values. D) Drug release properties against different drug loading concentrations (IMT: Imatinib). (C,D) Reproduced with permission.^[^ ^115^ ^]^ Copyright 2017, Elsevier.

Different factors can influence MBG drug release kinetics and cumulative release. In anticancer drug delivery, a carefully controlled and sustained drug release is crucial. Reducing MBGs pore size usually results in a lower drug release rate.^[^ ^116^ ^]^ Moreover, drug loading concentrations affect the release profile of MBGs.^[^ ^115^, ^118^ ^]^ In addition to drug loading, therapeutic ions and trace elements also affect drug release profiles. Example (1); the addition of selenium (Se), europium (Eu), and terbium (Tb) to MBGs has been shown to decrease drug release kinetics.^[^ ^36^, ^97^, ^112^, ^120^, ^121^ ^]^ It was shown that a larger surface area of Se/MBG causes more physical interactions between Se/MBG silanol (Si—OH) groups and DOX hydroxyl (—OH) and amine (—NH_2_) groups. Moreover, these interactions inhibit DOX transportation from the inner to the outer mesoporous structure to some extent.^[^ ^120^ ^]^ Example (2); Tb/MBG showed a lower drug release rate owing to higher specific surface area and pore volume, making drug incorporation into the inner mesopores easy.^[^ ^97^ ^]^ Also, highly hydrophilic Eu/MBG can quickly adsorb water‐soluble DOX molecules on their own charged surface and soak them into internal pores due to the stronger interactions of Eu/MBG and DOX. This feature increased the DOX loading and slowed down its release rate.^[^ ^121^ ^]^ It has been concluded that pH has a critical role in drug release kinetics; as the drug release environment's pH value decreases, the drug release rate increases leading to higher drug dosage in the acidic environment of cancerous tissues and cells (**Figure** **7**).^[^ ^36^, ^97^, ^112^, ^120^, ^121^ ^]^ Furthermore, studies concluded that MBGs doped with Eu, Tb, Se, and samarium (Sm) radionuclides led to a drug release mechanism governed by Fickian diffusion. The results were also compared with the prediction of the Higuchi model (*Q*_H_ = *k*_H_ × *t* ^1/2^, *Q*_H_ is the drug release amount at the time “*t*”, and *k*_H_ is the Higuchi dissolution constant).^[^ ^97^, ^111^, ^112^, ^120^, ^121^ ^]^

**Figure 8:**
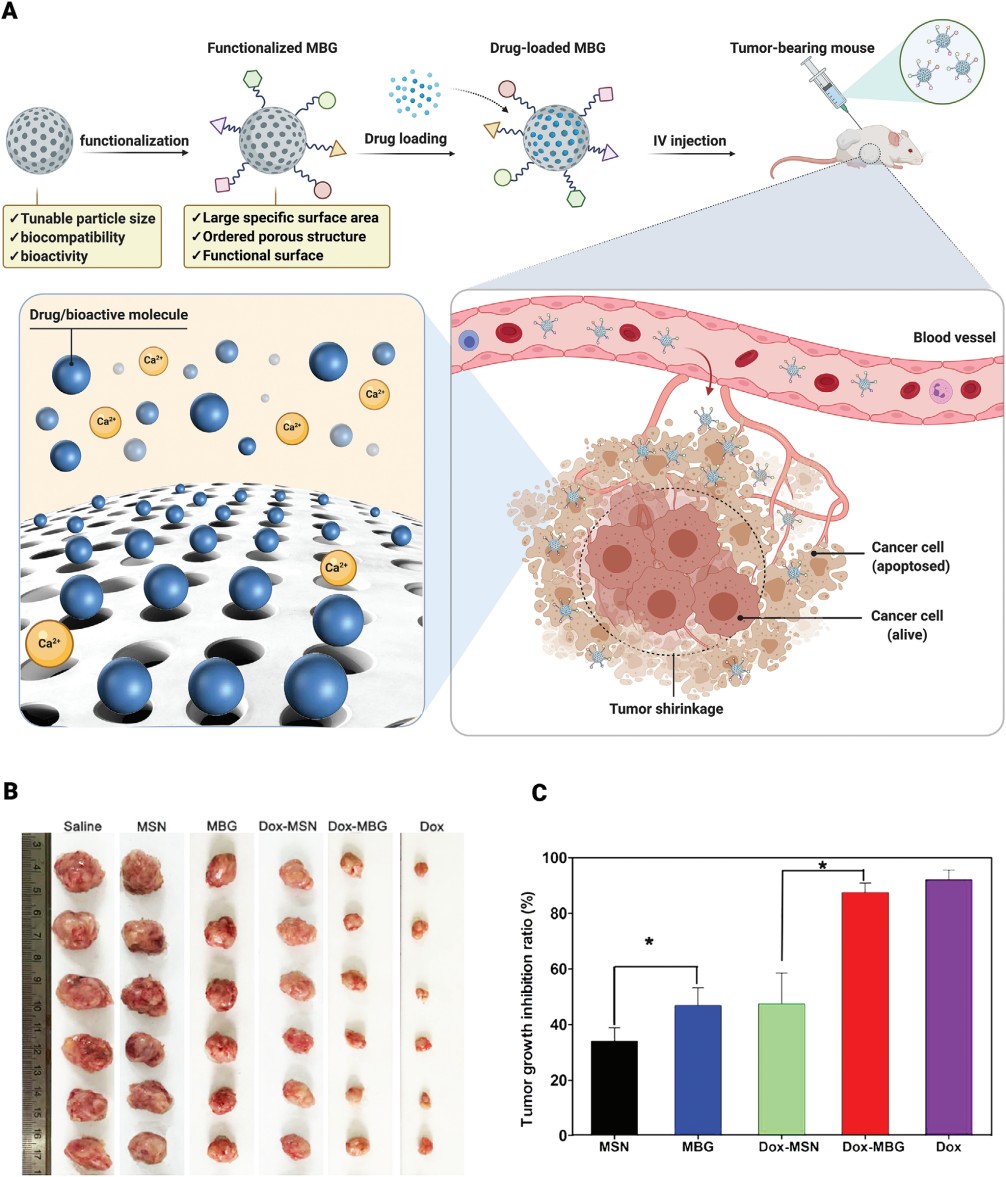
MBGs application for anticancer drug delivery in vivo. A) Schematic illustration of MBG nanosphere functionalization mechanism and drug loading and drug‐loaded MBG effect on tumor size. B) In vivo antitumor efficacy of dendritic MBG nanospheres in a mouse tumor xenograft model. Mice were injected with saline, mesoporous silica nanoparticles (MSN), dendritic MBG, DOX–MSN, DOX–MBG, and free DOX. Image shows the solid tumors removed at the end of the study. C) Tumor growth inhibition ratio. (IV: Intravenous; *: *p* < 0.05). Reproduced with permission.^[^ ^122^ ^]^ Copyright 2018, American Chemical Society.

To investigate MBGs in vivo antitumor effectiveness, prepared MBG nanospheres were established as functional drug delivery systems for drug loading and release analysis. Their influence on tumor inhibition in the in vivo tumor xenograft model, bio‐distribution, clearance, cellular location, and systemic toxicity were also evaluated to assess MBGs performance in vivo. Optimized MBG nanospheres exhibited a dendritic mesoporous structure with a large specific surface area, efficient drug loading and release in a controlled manner. This unique structure can effectively prolong the drug half‐life and suppress tumor growth without affecting normal cells. Furthermore, DOX‐loaded dendritic MBG nanospheres significantly decreased tumor volume in the murine S180 sarcoma model and reduced systemic toxicity (**Figure** **8**).^[^ ^122^ ^]^

#### 5.1.2 Radiopharmaceuticals Delivery

Low dose radiation in external radiotherapy is a prerequisite due to the effect of radiation on healthy tissues. In conventional radiotherapy, the X‐ray source is irradiated on the tumors from outside the body. If a high radiation dose is exploited, it can damage the surrounding healthy tissues. External radiotherapy's efficacy is restricted to low radiation dose, which reduces the possibility of receiving radiation to internal cancerous organs such as the kidney and liver. By contrast, for in situ or selective internal radiotherapy (brachytherapy), the radioactive isotope Yttrium (^90^Y), for example, as yttrium aluminosilicate glass, YAS, is the most investigated and popular substance which is implanted in cancerous tumors or injected into blood vessels to achieve the target site. This allows a higher dose of localized radiation to be transmitted to the target tissue and reduces healthy tissues’ damages.^[^ ^124^, ^125^ ^]^ Its deficits, such as poor biodegradability and bioactivity, let the authors investigate better substances. BGs have been introduced for this purpose owing to its bioactivity and biodegradability.^[^ ^126^ ^]^

In clinical applications, radioisotopes are bombarded by neutrons and activated before injection. If they are incorporated into microspheres for example made by MBG, the leakage of these radioisotopes will be reduced while the lifespan/biocompatibility of the radionuclide vector will be increased.^[^ ^125^, ^127^ ^]^ Moreover, the synthesized MBGs should not contain unwanted elements that could produce undesirable radioisotopes during the activation pathway.^[^ ^128^ ^]^ For the first time, Christie et al.^[^ ^125^ ^]^ used BGs as a radionuclide vector and incorporated yttrium in its structure to assess this composition's ability for in situ cancer therapy. They concluded that the yttrium release profile depended on the interplay between the yttrium ion environment and yttrium ion clustering behavior. They also showed that yttrium addition results in a higher release rate of ions due to less durability of the glass as the network connectivity decreases notably.

**Figure 9:**
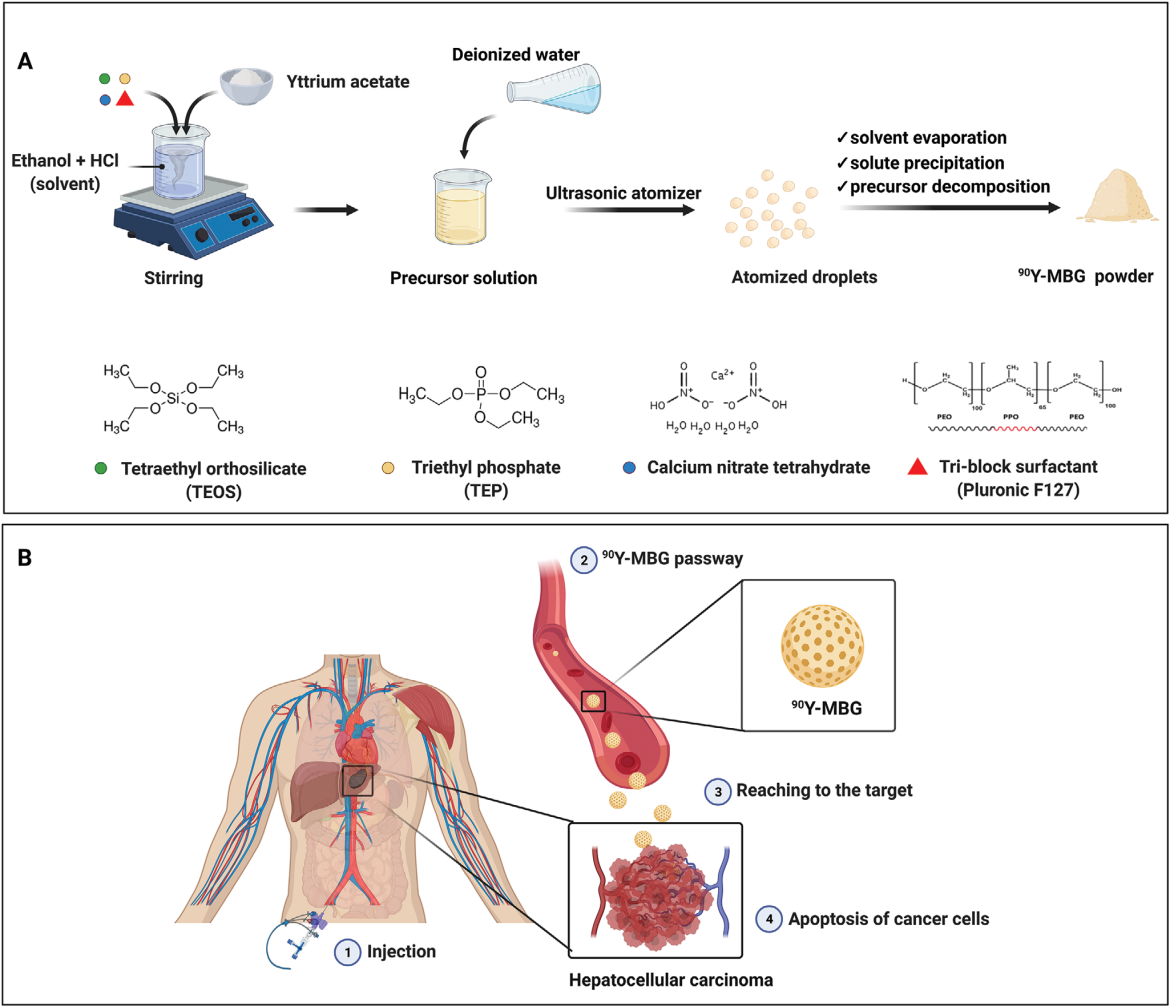
Schematic of selective internal radiotherapy (SIRT) via ^90^Y‐MBG for hepatocellular carcinoma. A) Synthesis of yttrium‐loaded mesoporous bioactive glasses (^90^Y‐MBG). B) Application of ^90^Y‐MBG for radiotherapy. A catheter is passed through the femoral artery and guided to arteries supplying the liver. Then, ^90^Y‐MBG is infused through the catheter into the arteries. When they land in the tumor, radiotherapy is performed, and the emitted radiation kills the cancer cells.

There is limited research on studying radioisotope‐loaded BGs or MBGs, mainly due to limited access to nuclear facilities.^[^ ^101^ ^]^ For instance, BG‐radiotherapy is exploited for the treatment of liver cancer. The microspheres of BGs (with a diameter of 25 mm) containing 50 (%w/w) Y_2_O_3_ are injected intravenously into patients to be placed in the capillary bed of the liver^[^ ^129^ ^]^ (**Figure** **9**). A 64 h half‐life was achieved for radioisotope‐activated microspheres capable of transmitting a dose of 15000 radians to malignant cells. This dose is significant compared to the maximum dose used in external radiotherapy (3000 radians).^[^ ^130^ ^]^ The radiation attenuation properties of BGs doped with NiO were also evaluated by determining various factors, including mass attenuation coefficient, exposure/absorption buildup factors. The doping of NiO in BGs demonstrates a substantial effect on photon interaction and high potency to attenuate *γ*‐radiation at medical therapy energies.^[^ ^131^ ^]^ Other researchers incorporated Samarium (^153^Sm) in a sol–gel synthesized BG and applied it for prostate cancer treatment via brachytherapy. The sol–gel method provided high porosity and the Sm radioisotope delivered more energy to the tumor tissue in a shorter half‐life )46.27 h) than Iodine (^125^I) (54.9 days).^[^ ^132^ ^]^

The present literature trend indicates that the full potential of BGs in brachytherapy is still to be deeply explored. It is expected that there will be further developments in this active area of research in the next few years.

### 5.2 Stimuli‐Responsive Nanoplatforms

Among various types of drug delivery systems applied in biomedicine, many of them just provide a sustained release of the cargo. The majority numbers of these systems have no capability to stop the payloads once it has begun. However, several diseases like cancer, diabetes, disorders related to hormones need a pulsatile release or spatiotemporal control to meet the patient's needs and also reduce the undesirable side‐effects. Stimuli‐responsive nanoscopical delivery can release cargos on‐demand as a response to external or internal triggers.^[^ ^133^, ^134^ ^]^ These smart systems can be activated through physiological triggers (endogenous) including pH, redox, enzyme, etc., external stimuli (exogenous) like light, magnetic or electric field, ultrasound, etc. and combination of the both stimuli. In the following sections, different stimuli‐responsive MBGs are showcased.

#### 5.2.1 Endogenous Stimuli

**Figure 10:**
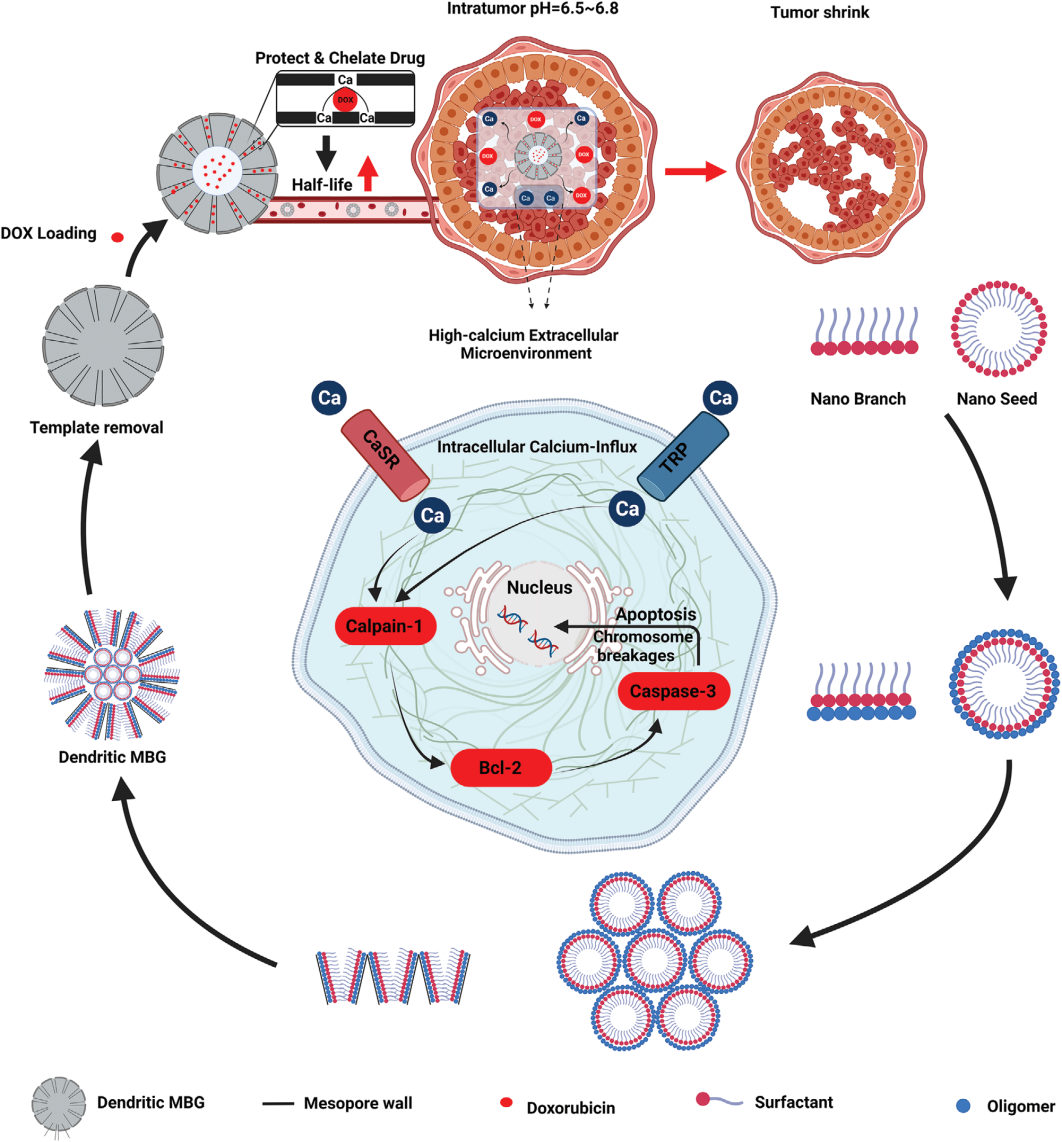
The simultaneous suppression effect of Ca ions and DOX molecules on the tumor growth (pH = 6.5–6.8). Schematic illustration of the development and tumor suppression of the pH‐sensitive DOX‐loaded dendritic MBG step by step.

pH‐responsive drug delivery systems contain ionizable moieties (carboxyl or amine) by which the structure changes in the exposure of different pHs. Speaking of cancer therapy, these systems are of particular interest as the pH of extracellular tumors and endosomes is ≈6.8 and 5.5 in turn. The driving force between the drug delivery system and the targeted site is the pH gradient stimulating the carrier to liberate its chemotherapeutic cargo in controlled manner.^[^ ^135^ ^]^ Up to now, a few studies focused on endogenous stimuli‐responsive MBG drug delivery systems for cancer therapy and they are mainly responsive to pH or enzyme. Amine‐functionalized MBG modified with maleic and cis‐aconitic anhydride was synthesized for bone cancer therapy. Maleic and cis‐aconitic as acid‐responsive materials belong to unsaturated dicarboxylic acid anhydrides and used in this study as a covalent bridge between the drug molecules and the MBGs surface functional groups.^[^ ^136^ ^]^ Nanosphere‐like DOX‐loaded MBG was synthesized through a facile hydrothermal synthesis technique as a multifunctional material for bone cancer therapy and regeneration. The nanospheres were not modified with any acid‐sensitive polymer, but showed a pH‐responsive characteristic when exposed to an acidic medium. The phenomenon was attributed to the dissolution of Ca from the nanospheres at acidic conditions as Ca played a chelating agent role in the MBG structure and interacted with DOX molecules.^[^ ^115^, ^137^ ^]^ Moreover, it has been demonstrated that the liberation of Ca ions from either MBG or BG occurring in a faster rate through acidic media is capable of inducing cell damage and so tumor suppression. Therefore, the acidic medium of tumor microenvironment not only increases the release rate of anticancer drug molecules, but also liberation of Ca ions, both of which simultaneously suppress the tumor growth (**Figure** **10**).

Aggregation‐induced emission luminogens were anchored on MBG to yield an organic‐inorganic pH‐sensitive drug delivery system. Regarding to strong blue emission of the luminogens, the obtained hybrid was endowed with luminescence property. The DOX‐loaded luminogens‐modified MBG was found to release the cargo faster at pH = 4.0 than 6.9 which was attributed to the lack of electrostatic interactions between the drug molecules and the substrate at the former pH.^[^ ^138^ ^]^ Besides using different organic modifiers to introduce pH‐sensitivity to MBGs, a study used hydroxyapatite to cap the drug‐loaded MBGs pores impeding the drug release at physiological conditions (pH = 7.4). As hydroxyapatite can be degraded through acidic media, the system showed a faster release at pH = 4.0 than the neutral pH.^[^ ^139^ ^]^ Recently, a pH‐responsive core–shell structure composed of poly‐l‐glutamic‐acid/MBG was designed and synthesized for release of a chemotherapeutic drug (daunomycin). A significant release was observed at the acidic condition, whereas a low leakage of drug molecules occurred at the physiological medium (pH = 7.4).^[^ ^140^ ^]^

**Figure 11:**
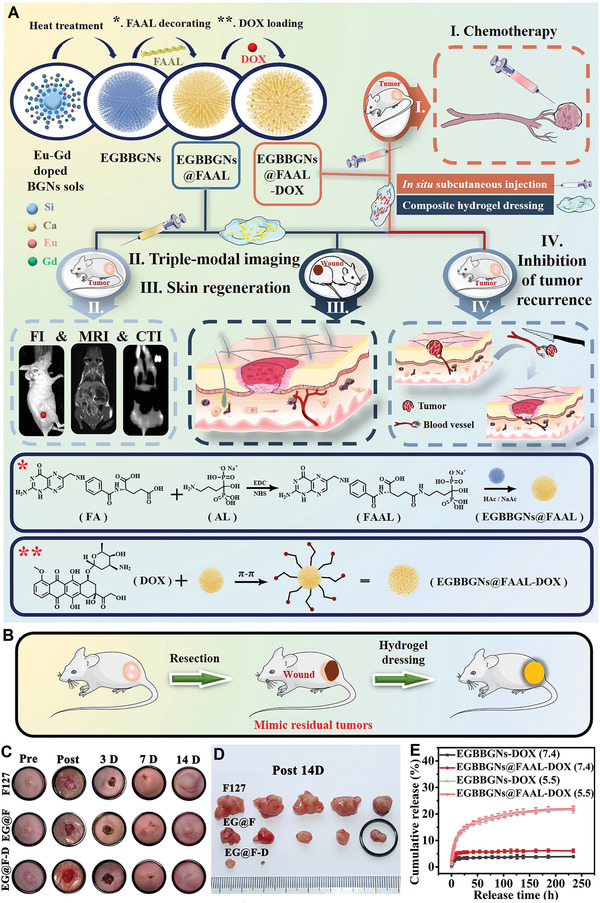
Multifunctional pH‐sensitive MBG for skin cancer therapy and regeneration. A) Schematic illustration of synthesis, decoration with folate‐alendronate (FAAL), and DOX loading of the mesoporous branched Eu‐Gd bioactive glass nanoparticles (EGBBGNs). The multifunctionality of EGBBGNs including imaging, melanoma therapy, and tissue regeneration. The interactions between the nanoparticles, surface modifiers, and DOX. B) Schematic on the inhibiting tumor recurrence. C) Photographs related to the wounds treated with different samples as follows: EGBBGNs‐FAAL (EG@F), EGBBGNs‐FAAL‐DOX (EG@F‐D), and F127 up to 14 days. D) The images related to the removed tumors of various samples. E) The release profiles of different samples at physiological and acidic media. Reproduced with permission.^[^ ^144^ ^]^ Copyright 2021, Elsevier.

The skin tissue regeneration gets tough when one of the most aggressive skin cancer types (melanoma) must be dealt with through surgery even though recurrence of the disease is probable. Normally, medical practitioners remove melanoma once it has been diagnosed as cancerous, and even some healthy tissues around the targeted tissue will be removed. This surgery will leave behind a chronic open wound susceptible to severe infection, requiring something potent to deal with it. It is worth mentioning that when an injury occurs through the skin tissue, there are four responses by the body to address the problem leading to regeneration. These responses include hemostasis to hamper the constant bleeding followed by deploying white cells in the injury site to stand against the potential infection (inflammation stage), proliferation, and tissue remodeling.^[^ ^141^ ^]^ All these steps take time to be completed. However, in the case of chronic wounds caused by diabetes, neither the body nor traditional wound dressings can solve the problem. That was the time for BG compounds to get involved with the precarious situation. Besides the blood coagulation and angiogenic properties of SiO_4_, CaO, and P_2_O_5_ oxides, the bioglass structure can be modified with other therapeutic ions, i.e., Ag, Zn, Cu, Mg, Co, etc., to accelerate the healing process and dealing with both inflammation and infection at the same time.^[^ ^142^, ^143^ ^]^ Recently, a theranostic multifunctional MBG decorated with alendronate and folate followed by being loaded with DOX was reported for skin cancer therapy, imaging, and regeneration. The modified MBG showed about 600 mg g^−1^ DOX loading which is ultrahigh plus pH‐responsivity at acidic media; there were two drug release stages including the burst release up to 24 h followed by a sustained release up to 120 h. A very low leakage (3.2%) was observed at the physiological pH (7.4), while the release ratio at pH = 5.5 increased significantly to 14.1% up to 24 h evidencing the pH‐sensitive nature of the MBG. The reason why such a phenomenon was observed ascribed to the protonation of —NH_2_ groups in the DOX structure. Both the in vitro and in vivo studies implied that the decorated MBG loaded with DOX efficiently eradicated the tumor through the DOX release and also prevented the tumor recurrence.^[^ ^144^ ^]^ The synthesis, multifunctional nature, drug release profiles, and in vivo studies related to the decorated MBG are indicated in **Figure** **11**.

Another type of endogenous stimuli‐responsive systems is based on the biocatalytic action of enzymes. The drug delivery systems modified with different types of enzymes work with the changes in the enzymes expression as overexpression of an enzyme can be a sign of pathological niche. Protease is known to break down peptides and proteins and its concentration through healthy tissues is very low, whereas through cancerous tissues it can be found high in quantity.^[^ ^145^ ^]^ The other types of materials applied in enzyme‐responsive cancer therapy are the enzyme cathepsin B,^[^ ^145^ ^]^ hyaluronic acid which is degraded by hyaluronidase enzymes in various cancers,^[^ ^146^ ^]^ azoreductase, and endopeptidase enzymes.^[^ ^147^, ^148^ ^]^ In the case of mesoporous silica nanoparticles, different types of molecular gates including enzyme‐responsive ones have been expansively used,^[^ ^149^ ^]^ but very few studies have worked on the enzyme‐responsive MBGs.^[^ ^32^ ^]^ A polyamine‐functionalized MBG loaded with an antibiotic drug and then capped with adenosine triphosphate was synthesized against bone infection. In the exposure of acid phosphatase, which increases in bone infections due to the bone resorption activation, the adenosine triphosphate's bonds undergo hydrolysis resulting in the faster release of the loaded drug molecules.^[^ ^32^ ^]^ In the case of cancer therapy, the same group developed a triamine‐functionalized MBG implemented with the same tailored molecular gates (adenosine triphosphate); the MBG was first functionalized with triamine followed by being loaded with DOX and finally capped with the adenosine triphosphate. In the presence of high levels of adenosine triphosphate, the molecular gates underwent hydrolysis and liberated the anticancer drug molecules. It is noteworthy that in the case of patients diagnosed with osteosarcoma, the serum adenosine triphosphate is found in higher contents.^[^ ^42^ ^]^

As mentioned before, the studies worked on stimuli‐responsive mesoporous silica nanoparticles are high in number, while a few studies have shed light on the endogenous‐stimuli‐responsive MBGs for cancer therapy. With great textural and regenerative properties of MBG in both soft and hard tissue, there can be innovative pathways to target different cancers with MBG‐based internal‐responsive drug delivery systems.

#### 5.2.2 Exogenous Stimuli

##### Magnetic‐Responsive MBGs: Hyperthermia Therapy

**Figure 12:**
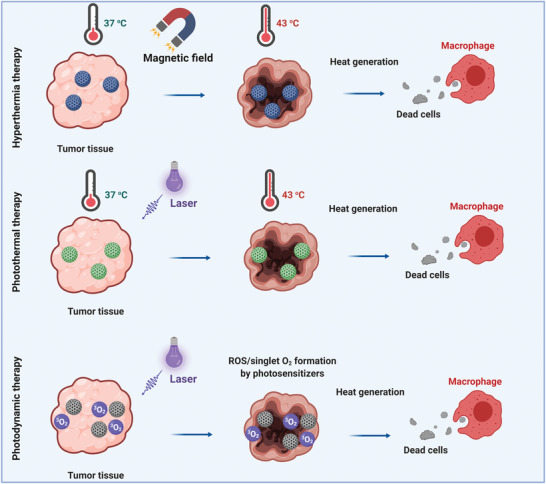
Eradication of cancerous tissues by different stimuli‐responsive MBGs. Schematic representation of magnetic‐mediated hyperthermia and photothermal and photodynamic therapy.

This class of MBGs typically pertained to hyperthermia therapy, a type of cancer therapy referred to cancerous tissues' eradication through released heat from an agent. Such materials are responsive to an EMF.^[^ ^150^, ^151^ ^]^ The heat generation capability of magnetic‐responsive nanomaterials can be altered with different parameters such as particle size, magnetization saturation, concentration, applied frequency, and magnetic amplitude of EMF.^[^ ^152^, ^153^ ^]^ Since the introduction of hyperthermia therapy, three heat ranges have been categorized under the umbrella of this technique: *T* ˂ 43 °C, 43 °C ˂ *T* ˂ 46 °C, and *T* > 46 °C. The first range (i.e., *T* ˂ 43 °C) is out of the present paper scope, and it has been applied in arthritis cases. The other ranges have been adopted to kill cancerous tissues. Temperatures above 46 °C are applied when a tumor ablation is required. It is critical to bear in mind that thermoablation has catastrophic effects on the adjacent tissues on the downside. At a temperature range of 43–46 °C so‐called mild hyperthermia occurs, which extirpates the cancerous cells and leaves the neighboring healthy tissues intact.^[^ ^154^ ^]^ **Figure** **12** depicts how different stimuli‐responsive agents can increase the local temperature of tumor tissue up to 43 °C under exposure to either an EMF or light.

**Figure 13:**
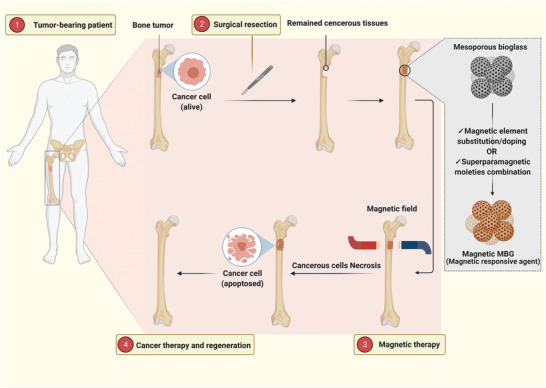
Treatment of a bone tumor through adopting magnetic‐responsive MBGs. The schematic shows how a magnetic‐responsive agent deals with remaining bone cancerous tissue after surgery.

Because of the many beneficial therapeutic and regenerative properties of MBGs for bone tissue, they have been mainly employed for simultaneous bone cancer therapy and regeneration. On the contrary to other types of cancers, bone cancer is more frequently diagnosed in youth than elders. Surgery is the very first response when a bone tumor is found. The tumor's removal leaves a defect, and the remaining surrounding tissue could increase the tumor revival risk.^[^ ^155^, ^156^ ^]^ Hence, the role of multifunctional stimuli‐responsive MBGs becomes evident. **Figure** **13** shows how a magnetic‐responsive agent can eradicate the remaining cancerous tissues under EMF exposure besides regeneration. MBGs are mostly composed of SiO_2_, CaO, and P_2_O_5,_ so they are not naturally regarded as magnetic. Therefore, the magnetic property should be introduced in the MBGs structure by combining with superparamagnetic moieties or substitution/doping of magnetic elements. Researchers in 2018 tried to turn a binary particulate‐like MBG composed of SiO_2_ and CaO into a magnetic BG through the addition of FeCl_3_ during the synthesis process. One of the hurdles to overcome was hampering some nonmagnetic phases, e.g., hedembergite (CaFeSi_2_O_6_) during the calcination process. As an interesting strategy, air and argon gas were applied to the calcination process and the results were compared. The argon gas led to the formation of ferrimagnetic phases (e.g., maghemite (*γ*‐Fe_2_O_3_) and magnetite (Fe_3_O_4_)), culminating in a significant increase in the magnetization saturation up to 2.17 emu g ^−1^. Argon gas was found to cause a narrower pore size distribution in the magnetic BG (26.4 down to 4.9 nm), which is beneficial for drug loading and controlled release over time.^[^ ^157^ ^]^ The same research group then turned the particulate‐like Fe‐doped MBGs into 3D bone scaffolds for bone cancer therapy and regeneration.^[^ ^158^ ^]^

A P123‐assisted sol–gel technique was adopted to synthesize MBG, Fe‐doped MBG, and FeCu‐doped MBG particles for hyperthermia‐based bone cancer therapy.^[^ ^159^ ^]^ This synthesis technique was shown to lead to composites with a high surface area. Interestingly, the composition containing both Fe and Cu elements outperformed the other samples in magnetization saturation. The reason behind this phenomenon was attributed to the effects of Cu addition on the glass structure, culminating in the superparamagnetic formation of Fe_3_O_4_ phase. Nonetheless, it is essential to notice that the introduced Fe in the glass structure can form three main phases of Fe_3_O_4_, *γ*‐Fe_2_O_3_, and *α*‐Fe_2_O_3_ (hematite), during the thermal treatment procedure. An enormous attraction was dedicated to Fe_3_O_4_ and *γ*‐Fe_2_O_3_ as intrinsic ferrimagnetic compounds, while *α*‐Fe_2_O_3_ was less attractive to researchers when a higher magnetization saturation was required.^[^ ^160^, ^161^ ^]^ *α*‐Fe_2_O_3_ has a transition temperature (Morin transition ≈ 263 K (−10.15 °C)) above which it becomes a ferrimagnetic material.^[^ ^162^ ^]^ A recent study has shed light on *α*‐Fe_2_O_3_ and investigated the growth of magnetic nanocrystals through the BGs structure for hyperthermia‐based bone cancer therapy.^[^ ^163^ ^]^ Different amounts (10, 20, 30 wt%) of *α*‐Fe_2_O_3_ were applied in the glass structure yielding a mesoporous magnetic glass–ceramic. Increase in the *α*‐Fe_2_O_3_ content up to 30 wt% was supposed to raise the nanocrystal size, and the crystal growth in this study was independent of the amount of Fe precursor. The addition of more Fe precursors led to an increase in the quantity of *α*‐Fe_2_O_3_ nanocrystals rather than the particle size. The obtained maximum magnetization saturation in this study was 3.49 emu g ^−1^. Besides the mentioned one‐pot synthesis routes based on the in situ formation of a ferrite‐based phase through the BGs structure, Ur Rahman et al.^[^ ^119^ ^]^ have applied a two‐step synthesis strategy including precipitation of Fe_3_O_4_ nanoparticles followed by addition to a P123‐assisted sol–gel synthesis system to yield a magnetic MBG for bone cancer therapy. The prepared MBG showed a magnetization saturation of nearly 15 emu g ^−1^ higher than values reported in previous studies.^[^ ^158^, ^159^, ^163^, ^164^ ^]^ The hyperthermia assessment indicated a sharp increase in the temperature up to 42 °C after 12 min, followed by a plateau until the ending point showing a saturated temperature.

There is an irreconcilable issue revolving around designing magnetic MBGs. On the one hand, the addition of iron is required to introduce magnetic property into the MBG structure and more iron has potential to increase magnetization saturation; the higher magnetization saturation, the more intense the heat generation. However, increasing in iron content is accompanied by a decrease in in vitro biocompatibility. Therefore, the lack of comprehensive studies shedding light on these challenges simultaneously can be felt. Notably, considering the available literature, it can be seen that the obtained magnetization saturation is mostly in the range of 1–4 emu g^−1^. Due to lack of in vivo studies in the available literature, it is still uncertain if the mentioned value is adequate to produce the required heat to eradicate cancerous cells. **Table** **3** summarizes the physical properties of applied magnetic‐responsive MBGs for simultaneous bone cancer therapy and regeneration (since 2018).

**Table 3:** Magnetic‐responsive MBGs with different compositions for simultaneous bone cancer therapy and regeneration

Sample/type	Composition	Magnetization saturation [emu g^−1^]	Surface area [m^2^ g^−1^]	Bioactivity/biocompatibility	Main results	Ref.
Fe‐doped MBG/particulate	60SiO_2_–(40‐*x*)CaO–*x*Fe_2_O_3_ (*x* = 2 and 10 mol%)	0.11–2.17	11.7–119.4	–/–	Fe_2_O_3_ in low contents did not cause devitrification, while 10 mol% yielded a glass–ceramic. The inert gas applied for calcination led to more magnetization saturation.	[157]
Fe‐doped MBG/scaffold	60SiO_2_–(40‐*x*)CaO–*x*Fe_2_O_3_ (*x* = 2 and 10 mol%)	–	16.5–80.2	Within 2 weeks immersion into SBF, the scaffolds’ surface was completely covered with newly formed apatite/–	The magnetic scaffolds with hierarchical porosity (macro‐ to mesoscale) were obtained. Increasing iron content decreased the surface area and pore size. Regardless of iron content, all scaffolds were highly bioactive in vitro.	[158]
CuFe‐doped MBG/particulate	68SiO_2_–23CaO–4P_2_O_5_–5Fe_2_O_3_ mol% 68SiO_2_–23CaO–4P_2_O_5_–5CuO mol% 68SiO_2_–18CaO–4P_2_O_5_–5Fe_2_O_3_–5CuO mol%	0.24–1.04	169.5–284.8	A sediment formation on the sample was occurred after 7 days soaking into SBF/no cytotoxicity was observed when 200 and 400 µg mL^−1^ concentrations had been applied against horse mesenchymal stem cells‐adipose	The CuFe doped MBG was found superparamagnetic. Addition of both Cu and Fe together led to an increase in surface area, magnetization saturation, and superparamagnetic property.	[159]
*α*‐Fe_2_O_3_‐doped BG/particulate	(100‐*x*)(58SiO_2_–33CaO–9P_2_O_5_)–*x*Fe_2_O_3_ (*x* = 10, 20, and 30 wt%).	1.91–3.49	–	The samples showed a bioactive behavior and calcium phosphate was precipitated on them upon soaking into SBF (7 days)/moderate cytotoxic effect was observed mainly due to iron oxide content against MC3T3‐E1 cells	Synthesis of a glass–ceramic containing hematite nanocrystals through a one‐pot sol–gel method. The glass‐ceramic was superparamagnetic with desirable bioactivity in vitro.	[163]
FeBa‐doped BG/particulate	(60‐*x*‐*y*)SiO_2_–36CaO–4P_2_O_5_–(*x*)Fe_2_O_3_–(*y*)BaO (*x* = 0, 10, and 15 mol%) (*y* = 0, 5, and 15 mol%)	0.04–2.27	–	–/–	The BaO–Fe_2_O_3_ bioactive glasses with particle size between 30 and 70 nm were synthesized. The heat generation ability of samples under an external magnetic field (300 kHz) was assessed in vitro. Up to 700 s, the Δ*T* (°C) has reached up to 60 °C.	[164]
Fe_3_O_4_–MBG/particulate	51SiO_2_–18CaO–20Na_2_O–4P_2_O_5_–7Fe_3_O_4_ mol%	14.16	309	The samples showed a bioactive behavior and calcium phosphate was precipitated on them upon soaking into SBF (7 days)/the cell viability of Fe_3_O_4_–MBG samples against normal human fibroblast and osteosarcoma cells were assessed and no toxicity was observed.	A multifunctional bioactive glass with hyperthermia and drug delivery potentials was synthesized. The hyperthermia effect of sample was assessed in the exposure of osteosarcoma cells under an alternating magnetic field for 20 min (250 kHz) and 70% decrease in the cell viability was observed.	[119]

##### Light‐Responsive MBGs: Photothermal and Photodynamic Therapy

All therapies categorized under a term called thermal therapy intend to raise a specific organ's temperature to a desirable point.^[^ ^165^ ^]^ Although increasing the temperature above 37 °C means a potential danger for the human body as the sign of fever with irreversible catastrophic effects on different organs, controlling over the temperature's increment would be accompanied by positive effects for cancer patients. Harnessing thermal treatment therapies goes back to the 19th century when scientists stumbled on a startling discovery. They injected living bacteria into the patients suffering from cancer. It turned out that a regression in the disease occurred stemming from an infection raised by the bacteria accounting for the occurrence of fever.^[^ ^166^ ^]^ Despite the popularity of magnetic‐responsive materials in cancer therapy, some other alternatives have also been explored to deal with the issue. Photosensitive materials have received significant attention in this field due to their capability of heat generation under laser radiation.^[^ ^167^ ^]^ leads to the development of photothermal therapy which used to be regarded ineffective and nonreliable resulting due to some disadvantages—simultaneous laser's energy absorption by both cancerous and healthy tissues accounting for damaging neighboring healthy tissues besides weakened heat generation.^[^ ^168^ ^]^ This technique has recently received considerable attention, and novel light‐responsive materials are involved in the process leading to a significant increase in the temperature locally with high efficiency.

**Figure 14:**
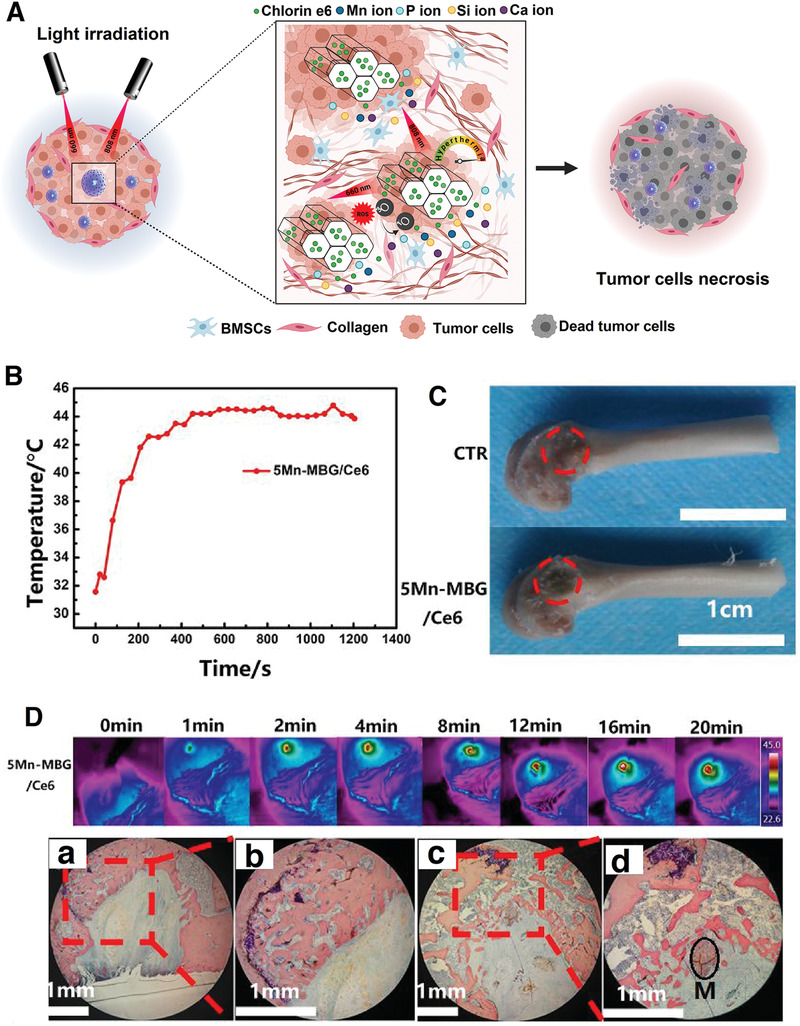
The combination of photothermal and photodynamic therapies for bone cancer therapy and regeneration. A) A schematic showing how Mn‐doped MBG/C6 works for bone cancer therapy and regeneration. B) The heating curve of 5Mn‐doped MBG/C6 after being implanted into critical‐sized femoral bone defects of rats. C) The defects photographs of control (CTR) and 5Mn‐doped MBG/C6 groups after 8 weeks. D) The thermal images after applying 808 nm laser irradiation up to 20 min to the 5Mn‐doped MBG plus Van Gieson staining images of (a,b) CTR and (c,d) 5Mn‐doped MBG/C6 at 8 weeks; the short‐term photothermal therapy had no significant negative effect on the bone regeneration. M represents the Mn‐doped MBG‐Ce6 particles. (B–D) Reproduced with permission.^[^ ^54^ ^]^ Copyright 2020, Elsevier.

There are numerous photosensitive agents applied for cancer therapy among which MoS_2_,^[^ ^169^ ^]^ gold nanoparticles,^[^ ^170^ ^]^ carbon‐based nanomaterials,^[^ ^155^, ^171^ ^]^ magnetic nanomaterials,^[^ ^172^ ^]^ LaB_6_,^[^ ^173^ ^]^ and CuFeSe_2_^[^ ^58^ ^]^ can be enumerated. Different approaches have been put forward to turn BGs into a light‐responsive agent. Dang et al.^[^ ^58^ ^]^ reported 3D‐printed BG scaffold's functionalization by semiconductor CuFeSe_2_ nanocrystals through in situ solvothermal technique. Another study deployed 2D ultrathin niobium carbide MXene nanosheets as a strong photosensitive agent for BG scaffolds.^[^ ^174^ ^]^ Another easy‐to‐use, innovative, and effective approach was adopted by Liu et al.^[^ ^165^ ^]^ through doping different metallic elements (Cu, Fe, Mn, and Co) into the BG structure. Besides the antibacterial properties of Cu and Co ions, they can induce angiogenesis, and Mn and Fe ions have stimulatory effects on bone mesenchymal stem cells.^[^ ^175^, ^176^, ^177^, ^178^, ^179^, ^180^ ^]^ In the case of light‐responsive MBGs, a recent study integrated both photothermal and photodynamic therapies into a single multifunctional platform composed of chlorin e6 (C6)‐loaded Mn‐doped MBG to present a potential solution for the eradication of deep bone tumors followed by an accelerated regeneration.^[^ ^54^ ^]^ The C6 is a photosensitive agent having the benefits of high singlet oxygen, and it has been applied in photodynamic therapies successfully.^[^ ^181^ ^]^ The combinational therapy underlaser radiation exposure was investigated regarding its effect on tumor growth inhibition. The heating curves were tracked carefully in vivo. It is visible that except for the pure MBG, the Mn‐doped MBGs revealed a dramatic rise in the temperature trends from around 25 up to 50 °C followed by a fluctuating trend in the range of 47–51 °C until the ending point (1200 s) (**Figure** **14**). Notably, the mild hyperthermia not only induced photothermal performance, but also triggered the C6 release leading to an improved uptake of C6 by tumor cells. Therefore, the photothermal performance enhanced the photodynamic therapy to achieve a more effective strategy for bone cancer therapy specially the deeper tumors.

**Figure 15:**
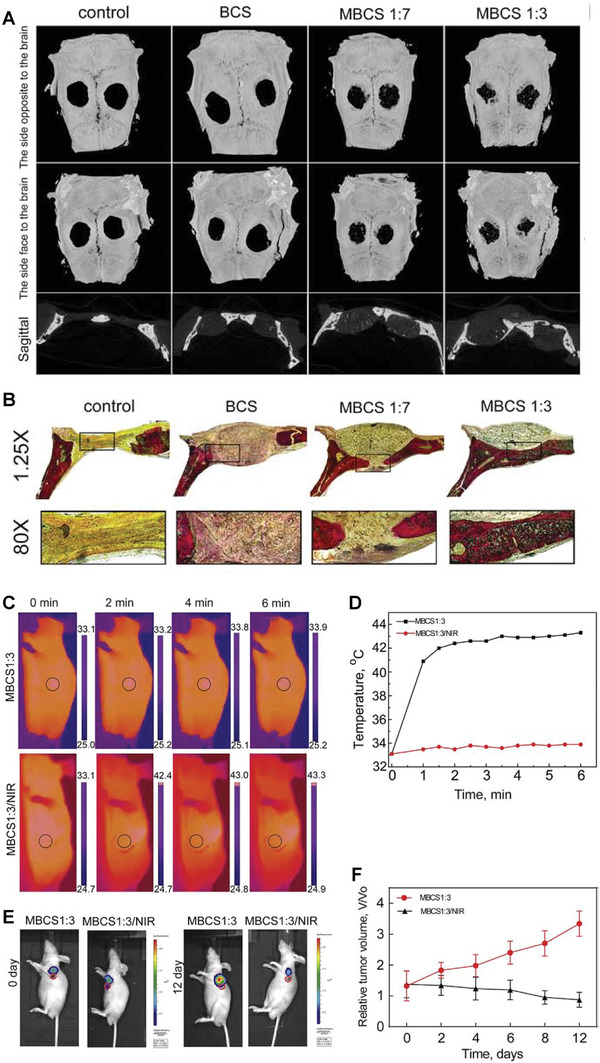
Light‐responsive SrFe_12_O_19_/MBG/chitosan composite scaffold for simultaneous bone cancer therapy and regeneration in vivo. A) Micro‐CT images of defects after being implanted with different samples; blank control, mesoporous BG/chitosan (BCS), SrFe_12_O_19_–BG–chitosan (MBCS) at two different ratios of 1:3 (MBCS1:3) and 1:7 (MBCS1:7). B) Light images relating to the Van Giesons picrofuchsin‐stained sections of defects filled with the scaffolds up to 12 weeks. The new bone tissue and scaffolds can be seen in red and black, respectively. C) IR thermal images and D) temperature (°C) versus time (min) curves of the implanted MBCS1:3 scaffold into tumor‐bearing mice with and without laser irradiation. E) Fluorescence images of implanted MBCS1:3 scaffolds into tumor‐bearing mice with and without laser irradiation up to 12 days. F) The change in tumor size over time after being treated with the MBCS1:3 scaffolds, *n* = 5. Reproduced with permission.^[^ ^172^ ^]^ Copyright 2018, Elsevier.

M‐type hexaferrite nanoparticles have both high magnetization saturation and high coercivity causing the preservation of magnetism even after turning off the EMF. This property has hampered their cancer therapy applications, where superparamagnetic nanostructures have always been preferred.^[^ ^182^ ^]^ However, being applied in the form of either a bulk or scaffold is entirely different from the particulate form. Lu et al.^[^ ^172^ ^]^ took advantage of this compound and fabricated a magnetic freeze‐dried bone scaffold. The scaffold was composed of SrFe_12_O_19_, MBG, and chitosan for simultaneous photothermal therapy and bone regeneration. Two mass ratios, including 1:3 and 1:7 for SrFe_12_O_19_/MBG, were applied and their tumor ablation efficiency was tested in vitro and in vivo. As the amount of SrFe_12_O_19_ increased, the heat generation ability of the scaffold rose as well. After being exposed to MG‐63 osteosarcoma cells, the scaffolds were triggered by laser radiation (808 nm, 4.6 W cm^−2^). This led to a sharp increase in the culture medium's temperature from 24 °C up to nearly 40 °C after 3 min of laser irradation. The test was continued for up to 6 min with the same conditions culminating in a significant decrease in cancerous cells' viability (**Figure** **15**). Moreover, the scaffold with 1:3 SrFe_12_O_19_/MBG mass ratio was implanted near a tumor tissue in vivo to assess its photothermal activity. A significant decrease in the tumor's volume after applying the laser radiation was obtained.

## 6 MBG and Proteins Interactions: Protein Corona

**Figure 16:**
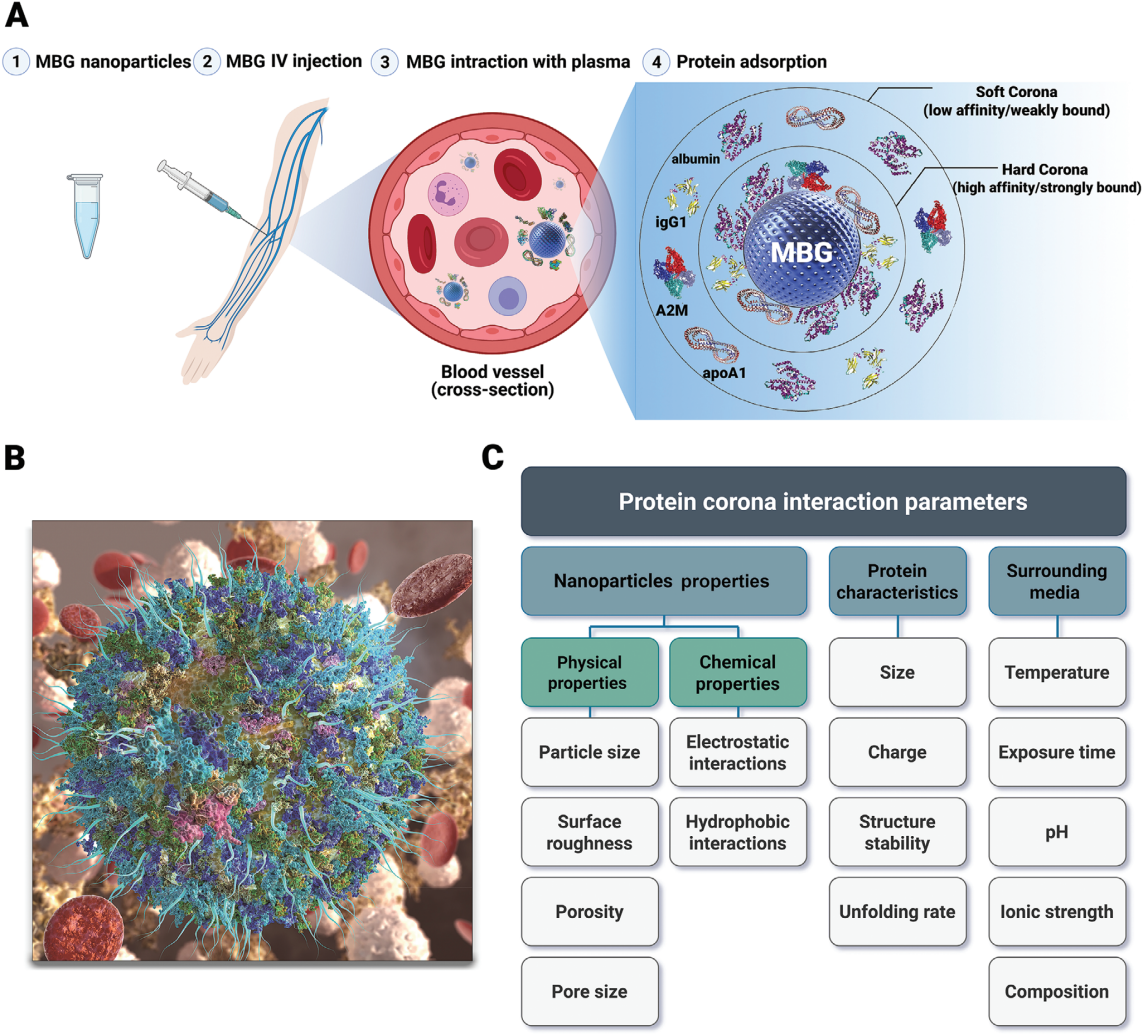
Protein corona formation and its determinant factors. A) Schematic illustration with a futuristic vision of the protein corona formation on MBG upon coming in contact with blood plasma. Reproduced with permission.^[^ ^208^ ^]^ Copyright 2014, American Chemical Society. B) Nanoparticle and protein corona formation (adsorbed blue, green, and cyan globules) upon nanoparticles contact with a biological fluid. Reproduced with permission.^[^ ^209^ ^]^ Copyright 2017, American Chemical Society. C) Diagram including major factors affecting protein corona formation; divided into three main categories. IV: Intravenous.

Proteins are complex biopolymers with a fundamental role in living organisms. Proteins conduct cell adhesion, proliferation, differentiation, and provide mechanical support besides having an essential role in mediating biological responses.^[^ ^183^ ^]^ Proteins are also superior biomaterials for biomedical applications, including drug delivery systems, coatings, and scaffolds for tissue engineering.^[^ ^184^, ^185^, ^186^ ^]^ Nanoparticles interact with various active biomolecules after coming in contact with body fluids.^[^ ^187^, ^188^ ^]^ Protein adsorption is the first event occurring after nanomaterials (e.g., MBGs) meet protein‐containing biological fluids (e.g., blood plasma) loading for the formation of a dynamic surface layer of proteins on pristine nanoparticles, called protein corona, playing a vital role in the interaction between the nanoparticles and the body.^[^ ^189^ ^]^ Protein corona formation is influenced by the nanoparticles' physicochemical characteristics, proteins properties, and surrounding media bioenvironment (**Figure** **16**).^[^ ^190^, ^191^ ^]^

The formation of the protein corona, as a complex phenomenon, becomes more distinctive with decreasing particle size. As the particle size reduces, the surface‐to‐volume ratio rises, nanoparticles show more surface activity and reduce their surface energy through interaction with biological fluids components, including proteins.^[^ ^192^ ^]^ Even though various parameters influence the formation and composition of the protein corona, this phenomenon becomes considerable when the particle size reduces to a certain extent (e.g., <100 nm).^[^ ^193^, ^194^ ^]^ Surface textures (e.g., mesoporous structure, porosity, and pore size) are a decisive factor determining the scale of surface areas that interact with the protein molecules. Surface texture variation results in alteration of surface‐related parameters, mainly specific surface area. In this regard, mesoporous structures possess enhanced protein adsorption mainly due to the enlarged specific surface area providing more active binding sites for proteins.^[^ ^195^, ^196^, ^197^ ^]^ Meanwhile, pore size sieves protein molecules penetrated to the pores and larger pore sizes facilitate adsorption of proteins with high molecular weight (e.g., bone morphogenic protein); therefore, pore size determines protein adsorption in a protein size‐dependent manner.^[^ ^198^, ^199^ ^]^

Nanoparticles chemical properties can also influence protein corona formation through electrostatic or hydrophobic interactions. Electrostatic interactions can either enhance or suppress protein adsorption determined by the chemical nature of nanoparticle surface and types of protein.^[^ ^200^ ^]^ Generally, proteins have more affinity to be adsorbed onto hydrophobic surfaces, although some proteins with massive hydrophilic domains (e.g., glycoproteins) have limited adsorption to hydrophobic surfaces. For instance, BG hydrophilicity is dependent on surface topography and chemical composition; however, BG surfaces are often highly hydrophilic. In general, BGs with lower hydrophilicity are expected to adsorb a greater number of proteins since fewer water molecules have to be expelled from glass surfaces prior to protein adsorption.^[^ ^199^, ^201^ ^]^

Proteins features (e.g., type, size, and hydrophilicity) can also impact the protein corona formation. Larger proteins have more binding sites to interact with solid materials surfaces being more susceptible to be adsorbed on the surface.^[^ ^202^ ^]^ Molecules near their isoelectric point often show higher surface activity and adsorb more readily.^[^ ^203^ ^]^ Proteins unfolding exposes more sites for the contact between protein and surface. Less stable proteins are more likely to unfold, forming more contact points with surfaces. For instance, lipoproteins are structurally unstable and thus show a strong affinity to hydrophobic surfaces.^[^ ^204^ ^]^ Furthermore, numerous proteins have active interplay together in addition to their interaction with the surface of the biomaterial. Proteins can have cooperative and/or competitive situations that can influence their surface adsorption.^[^ ^189^, ^204^ ^]^

The amount of protein adsorption can be influenced by other parameters in the surrounding medium available in biological fluids, including temperature, exposure time, dissolved ion concentrations (ionic strength), pH, and surrounding medium composition. Temperature elevation generally increases adsorbed protein concentration, and higher ionic strength enhances protein aggregate tendency. For instance, acidic pH values have been shown to cause more protein adsorption amounts on BG surfaces.^[^ ^204^, ^205^, ^206^ ^]^ A longer duration of exposure time increases the total protein quantity adsorbed. Furthermore, the initial formation of the corona is called the soft corona, which includes reversible, loosely bound proteins and has a short half‐life. Soft corona develops with time to hard corona, consisting of proteins with a strong affinity for the nanoparticle surface, forming an irreversible tightly attachment between the protein and the surface of the nanoparticles.^[^ ^207^ ^]^

**Figure 17:**
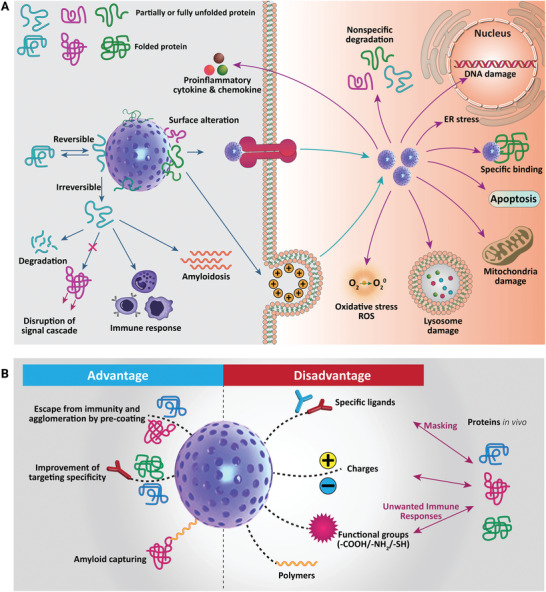
Extracellular and intracellular reactions due to structural changes of the protein corona. BG–protein interaction causes various signal modulations and toxic effects in biofluids and cells. Reversible and irreversible orientation and conformational changes of protein structure after adsorption can perturb downstream signaling that may be harmful to the host. Protein corona formation has different extracellular and intracellular effects. B) The pros and cons of protein corona formation. ER, endoplasmic reticulum; ROS, reactive oxygen species.^[^ ^212^ ^]^

Protein corona alters the characteristics of nanoparticles compared with bare nanoparticles. This phenomenon induces a new biological profile that affects nanoparticle interaction with the cell membrane, biodistribution, and toxicity in biological fluids.^[^ ^192^, ^210^, ^211^ ^]^ The composition, amount, and formation of adsorbed proteins affect the biocompatibility of biomaterials. Protein corona formation can decrease cytotoxicity and enhance biocompatibility. On the other hand, it can cause adverse responses such as thrombosis and infection due to uncontrolled protein adsorption and subsequent reduction in biocompatibility. This aspect is considered a result of orientation and conformational changes of proteins due to adsorption to a solid surface.^[^ ^212^, ^213^, ^214^, ^215^ ^]^ Furthermore, the cell adhesion and attached cells’ morphology and motility are also dependent on adsorbed proteins on the nanoparticles surface.^[^ ^189^, ^212^ ^]^ Extracellular and intracellular interactions due to the protein corona's structural changes are further explained in **Figure** **17**.

MBGs are applied in anticancer therapeutic approaches owing to their drug delivery potential. Despite extensive in vitro investigation of these nanoparticles for active and passive anticancer targeted therapy, in vivo tumor targeting ability and therapeutic efficiency of these platforms is still a challenge leading to few clinical trials and even fewer clinical practices. Between various mechanisms, including modified physiological environment of tissues and organs and physical barriers persistence, protein corona formation plays a vital role in impairing targeted delivery, sustained drug release, and subsequent low therapeutic efficiency.^[^ ^122^, ^216^, ^217^, ^218^ ^]^

Protein corona formation affects both active and passive drug delivery mechanisms. Protein accumulation buries nanoparticles targeting ligands such as antibodies, aptamers, and peptides resulting in limited accessibility of targeting ligands to cell receptors and consequent loss of targeting to the tumor site. Protein corona formation decreases nanoparticles leaking to the tumor site, diminishing the efficiency of the passive drug delivery platform.^[^ ^219^ ^]^ Dependent on the nature of the adsorbed protein, the protein corona can enhance or decrease nanoparticles susceptibility to clearance by the systemic circulation through phagocytosis by the mononuclear phagocyte system. Accumulation of opsonin proteins, such as fibrinogen and immunoglobulin G, on nanoparticles surface, enhances their recognition and ingest by macrophages resulting in prompt clearance from systemic circulation. Antithetically, protein corona composed of dysopsonin proteins, such as albumin and apolipoproteins, enhance nanoparticles' systemic circulation.^[^ ^109^ ^]^

Effective anticancer drug delivery is achievable by a proper protein corona design, and for this purpose, various strategies have been investigated based on three main approaches. The biomimetic approach uses different ligands to elude clearance by the mononuclear phagocyte system, thereby enhancing their systemic circulation. For instance, A*β*_1‐42_ (SP) biocompatible peptide, which can interact with apolipoproteins, has been employed for liposome surface functionalization. This designed liposomal system potentially adsorbs plasma apolipoproteins, especially Apolipoprotein E, when exposed to biological fluids. The functionalized liposomal system showed better distribution and enhanced drug delivery efficacy compared to the plain liposomal system.^[^ ^220^ ^]^ Nanoparticle functionalization with hydrophilic polymers such as polyethylene glycol, polyvinylpyrrolidone, and dextran is achieved to inhibit protein corona formation. This system increases systemic circulation of the nanoparticles prior to reaching target sites owing to macrophage uptake avoidance.^[^ ^109^ ^]^ As mentioned earlier, hard protein corona formation alters nanomaterial's identity. Accordingly, employing specific artificial protein corona (protein corona shield) on nanoparticles that could inhibit adsorption of undesired proteins such as opsonin protein can be considered a promising strategy to elude the immune system and increase the targeting ability of nanoparticles. For example, recombinant fusion protein consisted of HER2‐binding affibody combined with a glutathione‐S‐transferase create a functional protein corona shield with minimal reactivity to proteins in physiological fluids.^[^ ^221^ ^]^

In addition to target delivery impairment, protein corona affects drug sustained release. It is concluded that protein corona significantly decreases the drug release burst effect, which plays a key role in controlled drug release and reduces the systemic adverse effect of anticancer drugs.^[^ ^219^, ^222^ ^]^

Protein corona formation on MBG surfaces is a complex process influenced by a variety of factors. As mentioned earlier, surface characteristics, protein properties, and environmental factors are influential in determining the composition, amount, and activity of the adsorbed protein.^[^ ^189^ ^]^ Despite the significant role of protein corona in defining nanoparticle drug delivery and release characteristics, there is a lack of studies on MBGs as a specific class of BGs with promising potential in anticancer drug delivery. Hence, several purposeful, well‐designed in vitro and in vivo studies with particular attention to MBGs distinct protein adsorption characteristics compared with conventional BGs are required to specify the influence of protein corona on MBGs and drug interactions.

## 7 Immunogenicity

MBG nanoparticles have gained increasing attention in nanomedicine, mainly due to their in vitro therapeutic potential as drug delivery systems (DDSs), for bone cancer treatment, and for tissue regeneration.^[^ ^19^, ^46^, ^223^ ^]^ Nevertheless, understanding the impact of MBG‐based platforms on the immune system and inflammation would be of crucial relevance to assess the biocompatibility of these nanomaterials, and thus to tune the interplay with immune cells.

Before discussing the effect of MBGs on immune response, the immune system and its function are introduced and briefly described. The immune system comprises entire tissues, organs, proteins, and a variety of specialized cells that cooperate in a fascinating way to mount an immune response. The immune system can defend against disease‐causing microbes or foreign materials, and it can also distinguish “self” from “nonself” antigens or “normal” from “abnormal” cells. The immune response is broken down into innate (nonspecific) and adaptive (acquired or specific) immunity. The innate immune system provides the first line of the host's defense through mechanisms that include physical/chemical barriers and effector cells, like natural killer (NK) cells, granulocytes, monocytes/macrophages, innate lymphoid cells, and endothelial/epithelial cells. They participate in phagocytosis and control of inflammation by producing cytokines and soluble mediators. The adaptive immune response is mainly carried out by two different classes of lymphocytes, T and B lymphocytes, the principal actors of the cell‐mediated and humoral immunity, respectively.^[^ ^224^ ^]^ At the bridge between innate and adaptive immunity reside dendritic cells (DCs), considered professional antigen‐presenting cells (APCs). Immature DCs patrol the periphery, where they regularly uptake and process self‐antigens, keeping self‐tolerance. Detection of invading pathogens or “danger” signals through pattern‐recognition receptors (PRRs) induces functional maturation to immunogenic DCs. It results in the expression of costimulatory molecules, the release of proinflammatory cytokines, upregulation of major histocompatibility complex (MHC) molecules that enhance the ability of DCs to migrate toward secondary lymphoid organs. In these organs, APCs interact with and present antigen coupled to MHC to naïve T lymphocytes that differentiate into antigen‐specific effector T cells. By contrast, B lymphocytes differentiate into cells secreting antigen‐specific antibodies (described in a treatise by Kaufmann^[^ ^225^ ^]^).

When engineered nanostructures enter the body as foreign materials, they likely encounter and interact with the innate immune system first. Following detection through PRRs, innate phagocytic cells, mainly APCs, engulf NPs, and produce inflammatory cytokines and a high level of reactive oxygen species (ROS). Chronic inflammation and sustained oxidative stresses might contribute to malignancy development and tumor progression, fueling cancer.^[^ ^226^ ^]^ Depending on their physicochemical features, nanoparticles can differently modulate immunogenicity, and they might trigger undesired immune responses.^[^ ^227^ ^]^ Therefore, nanomaterial immunotoxicity needs to be addressed in both in vitro and in vivo studies to assess the suitability of these nanomaterials for nanomedicine applications.

**Figure 18:**
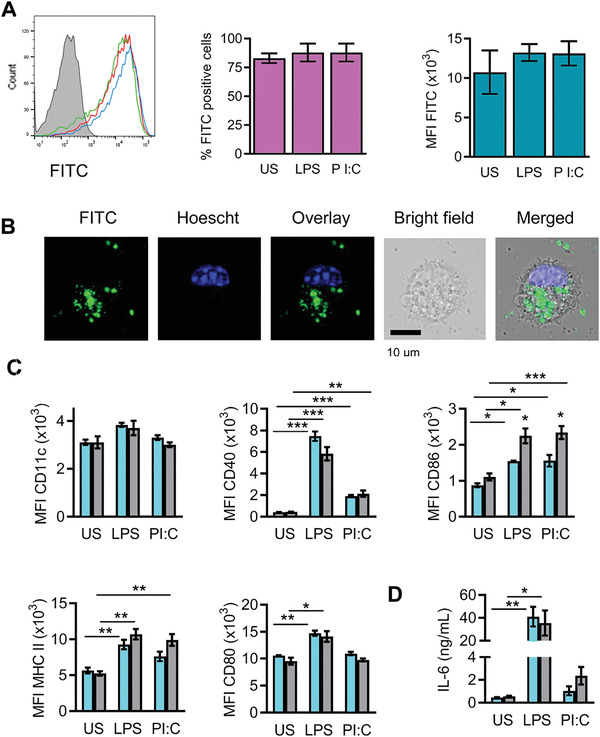
Uptake of MBG nanoparticles by bone marrow‐derived dendritic cells (BMDCs). A) Flow cytometry analysis of unstimulated (green, US), or LPS (red) or Poly I:C (PI:C, blue) stimulated BMDCs treated or untreated (gray) with fluorescein isothiocyanate (FITC)‐labeled‐MBG nanoparticles during 24 h. Graphs show the percentage of FITC^+^ cells (middle panel) or the median of fluorescence intensity (MFI, right panel). Immature as well as mature BMDCs take up the nanosphere. B) Confocal microscopy analysis of BMDCs after incubation of 2 h with FITC‐MBG nanoparticles shows the cytosolic distribution of nanospheres. FITC‐MBG nanoparticles are shown in green, in blues is shown cell nucleus stained with Hoescht dye. C) Unstimulated, or LPS‐ or Poly I:C‐stimulated BMDCs were incubated (gray bars) or not (light blue bars) with nanospheres during 24 h and evaluated for the expression of CD11c, CD40, CD86, MHC II, CD80 surface markers. Graphs show the MFI. MBG nanoparticles do not alter the maturation status of BMDCs, except the expression of CD86. D) ELISA assay for the detection of IL‐6 in 24 h culture supernatants of BMDCs incubated (gray bars) or not (light blue bars) with nanospheres, in the presence or without stimuli. MBG nanoparticles do not induce the proinflammatory IL‐6 cytokine in immature BMDCs (US), indicating that these nanomaterials do not induce DC maturation by themselves. Reproduced under the terms of CC‐BY license open access.^[^ ^229^ ^]^ Copyright 2020, MDPI.

Although MBG design and development as DDS dated back to 2004, the immunological profile of this platform has been argued only lately. Montes‐Casado and co‐workers recently addressed the in vitro compatibility of mesoporous SiO_2_–CaO nanospheres (NanoMBGs) on immune cells involved in both innate and adaptive immune responses. MBG nanoparticles were efficiently internalized by endocytic pathways, involving actin, clathrin, and phosphoinositide 3‐kinase (PI3K) activity, into different murine cells, as spleen B (CD19^+^) and T (CD4^+^ and CD8^+^) cell subsets or bone marrow‐derived dendritic cells (BMDCs).^[^ ^228^ ^]^ Cell activation and maturation status is not altered, and with only a slight increase in CD86 expression in LPS‐ or Poly I: C‐stimulated mature BMDCs. Neither splenocyte frequencies nor cytokine production was affected by MBG nanoparticles, including secretion of the inflammatory IL‐6 cytokine, overall underscoring their biosafety profile (**Figure** **18**).^[^ ^229^ ^]^

**Figure 19:**
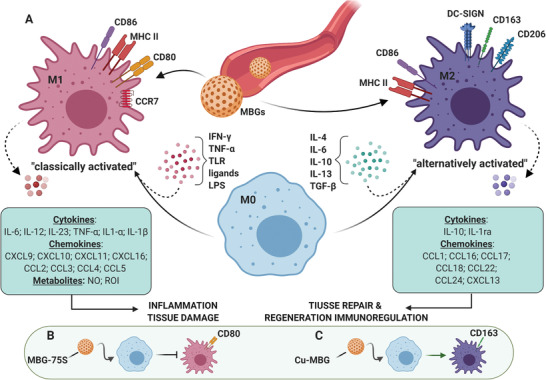
A simplified view of activation and functional polarization of macrophages in response to MBG nanoparticles. A) Figure summarizes selected features of macrophages polarized toward the proinflammatory M1 or anti‐inflammatory/prohealing M2 phenotype. Depending on different microenvironmental cues, uncommitted (M0) macrophages undergo either classical (e.g., IFN‐*γ* + LPS or TNF) or alternative (e.g., IL‐4/IL‐13) activation, acquiring distinct phenotypic and functional properties. Cytokine release profile of M1‐polarized cells includes IL‐6, IL‐12, IL‐23, TNF‐*α*, IL‐1*β*, as well as NO and ROI. Cytokine release profile of M2‐polarized cells includes IL‐4, IL‐10, IL‐13, IL‐1ra. Induction and activation of M1 cells lead to inflammation and tissue damage, while M2‐polarized cells promote tissue repair and regeneration. B) Phagocytosis of MBG‐75S nanomaterials does not induce macrophage polarization toward the proinflammatory M1 phenotype. C) In vivo phagocytosis of Cu‐MBG nanoparticles by monocyte/macrophages leads to their activation and polarization into the anti‐inflammatory CD163^+^ M2 phenotype. IFN‐*γ*: interferon‐gamma; LPS: lipopolysaccharide; TNF‐*α*: tumor necrosis factor‐alpha; TLR: toll‐like receptor; TGF‐*β*: transforming growth factor‐beta; NO: nitric oxide; ROI: reactive oxygen intermediate.

Macrophages are a heterogeneous population of phagocytic cells playing a central role in inflammation. In response to several environmental stimuli, they may exhibit different phenotypes and functions, displaying a significant degree of plasticity. Macrophages are classified into 1) resting or naïve M0 macrophages (M2‐like cells), 2) classically activated type 1 proinflammatory or M1 macrophages, 3) or alternatively activated type 2 anti‐inflammatory or M2 macrophages. M1 cells produce proinflammatory cytokines and reactive nitrogen and oxygen intermediates, promoting inflammatory responses and mediating tissue damage. By contrast, M2 cells (or M2‐like cells) generate anti‐inflammatory cytokines and tissue‐regenerative factors, orchestrating repair and retaining homeostasis. Therefore, macrophage plasticity is critical for the resolution of chronic inflammation.^[^ ^230^ ^]^ In many solid tumors, tumor‐associate macrophages (TAMs) are the most frequent immune cells infiltrating the tumor microenvironment (TME). They play important roles in tumorigenesis, by secreting cytokines, chemokines, growth factors, enzymes, and are involved in resistance to cancer chemotherapy and radiotherapy. Likewise, TAM can be classified as M1 and M2 macrophage cell subtypes. In general, they are thought to closely resemble the M2 macrophages,^[^ ^231^ ^]^ expressing among the other surface markers CD163, CD206, and Arginase‐1 (**Figure** **19**), although recent results provide evidence of an intermediate state of TAMs.^[^ ^232^ ^]^ NP‐based strategies aimed at eliminating, re‐educating or targeting M1 macrophages in inflammation and M2 macrophages in cancer are amidst the desirable approaches to be pursued.^[^ ^233^ ^]^ Some works have been carried out to evaluate MBG nanoparticle effect on macrophage polarization in this context.^[^ ^234^, ^235^ ^]^ An in vitro study focused on the effect of an MBG nanoparticle with molar composition 75SiO_2_–20CaO–5P_2_O_5_ (MBG‐75S) on macrophage polarization toward the proinflammatory M1 phenotype. In this regard, a mouse macrophage cell line was cultured for 24 h with or without 1 mg mL^−1^ of MBG‐75S, in the presence or absence of inflammatory stimuli, and evaluated for the expression of the M1 marker CD80. Compared to the control, MBG‐75S treated macrophages did not exhibit the M1 phenotype, indicating that these nanomaterials would trigger innate immune responses without leading to chronic inflammation.^[^ ^236^ ^]^ In vivo implantation of MBG incorporating Cu^2+^ inorganic ions (Cu‐MBG) recruited more anti‐inflammatory CD163^+^ (M2 marker) macrophages than the MBG control, without any significant difference in the expression of the M1 marker CCR7 between groups of animals receiving MBG or Cu‐MBG implants. By contrast, in the in vitro assay, the expression levels of IL‐1*β* (M1 marker) and IL‐1ra (M2 marker) increased after Cu‐MBG exposure.^[^ ^237^ ^]^ The authors speculated that the difference observed between the in vivo and in vitro data was due to the complexity of the in vivo microenvironment and overall suggested that Cu‐MBG may trigger tissue‐regenerative macrophages.^[^ ^237^ ^]^ Altogether, these results indicate that MBG nanomaterials might polarize macrophages toward the M2 phenotype which is more favorable for tissue regeneration and inflammation resolution (Figure 19).

To reduce in vivo inflammation, MBG nanoparticles might be formulated with antioxidant agents to lower the expression of pro‐inflammatory and oxidative stress‐related genes.^[^ ^226^ ^]^ Accordingly, MBG nanoparticles containing the antioxidant cerium (Ce) element appeared to be capable of downregulating the expression of IL‐1*β* and IL‐6 proinflammatory genes in macrophages cultured in vitro with Ce‐MBG nanoparticles. Such nanoparticles can also minimize the oxidative stress responses induced in the presence of oxidizing agents. Besides, Ce‐MBG nanoparticles were confirmed to not exert cytotoxicity against fibroblasts, assessing their in vitro biocompatibility.^[^ ^238^ ^]^ Recently, the incorporation effect of boron (B) into MBGs on inflammatory response was investigated. The results indicate that boron‐doped MBGs were not cytotoxic toward mouse bone marrow stromal cells at concentrations of 0.1–1 mg mL ^−1^. When macrophages were incubated for 4 h with boron‐doped MBGs or MBGs at a concentration of 1 mg mL ^−1^, the presence of boron significantly decreased IL‐6 gene expression compared to macrophages incubated with boron‐free MBGs. This mechanism unfolds the ability of boron to reduce inflammatory responses upon MBG administration.^[^ ^35^ ^]^ Overall, these studies confirm the biocompatibility of MBG nanoparticles as they do not trigger harmful immune responses.

Among the next‐generation antitumor approaches, immunogenic cell death (ICD) may be considered as a promising strategy to be pursued for treating and likely eradicating cancer. ICD is a form of regulated cell death that triggers adaptive immune cells to mount an antitumor immune response, eventually generating long‐term immunological memory.^[^ ^239^ ^]^ This process occurs when dying malignant cells mediate immunostimulatory effects in response to specific stress via the release of cytokines and adjuvant‐like signals and through the expression of tumor neoantigens that reshape the immunosuppressive TME into an immunogenic microenvironment. These events collectively support the recruitment and the activation of APCs which cross‐prime tumor‐specific cytotoxic T lymphocytes into draining lymphoid organs, potentially driving the tumor‐targeting immunity at microenvironmental level.^[^ ^239^ ^]^ Several stimuli and anticancer treatments can lead to the ICD cascade by triggering ROS generation that induces the intracellular endoplasmic reticulum (ER) stress or through the mitochondrial outer membrane permeabilization phenomenon.^[^ ^240^, ^241^ ^]^ Chemotherapeutic drugs (e.g., DOX), oncolytic viruses, physicochemical therapy, radiotherapy, PTT, irradiation, and PDT are considered as bona fide ICD triggers.^[^ ^240^, ^242^ ^]^ When tumor cells undergo ICD, they expose or secrete and release a panel of immunogenic damage‐associated molecular patterns as cellular adaptation in response to the stress. These include the cell‐surface ER calreticulin (CRT) and the heat‐shock proteins (HSP70 and HSP90), the secreted adenosine triphosphate, the released high‐mobility group box‐1, the mitochondrial DNA (mtDNA), cytokines as type I interferons (IFNs) and IL‐1 family members.^[^ ^241^, ^243^ ^]^ While conventional ICD approaches might suffer from some limitations (e.g., limited accumulation and retention, drug resistance, toxicity, and slow release in tumor tissue and cancer cells), nanosized DDSs might be employed to overcome these challenges and to improve cancer immunotherapy through ICD boosting.^[^ ^244^ ^]^ Currently, diverse lipidic, polymeric, and inorganic DDSs engineered for the delivery of chemotherapeutic ICD inducers are under investigation.^[^ ^242^ ^]^ Additionally, nanomaterials‐based PDT combined with PTT, chemotherapy, radiotherapy, and immunotherapy have been shown to synergistically enhance ICD.^[^ ^245^ ^]^ Considering the great potential of MBGs as DDS and/or stimuli‐responsive platform, it is reasonable to suppose that the bona fide ICD agents embedded into MBG nanoparticles are able to exert the immunogenic death of cancer cells. For instance, zinc‐containing BGs administered to MG‐63 human osteoblasts cell line were demonstrated to be able to induce in a dose‐dependent manner the release of markers indicative of oxidative stress.^[^ ^246^ ^]^ Similarly, holmium‐containing BGs inserted in a thermoresponsive hydrogel activated selective osteosarcoma cell death by inducing reactive oxidizing agents.^[^ ^247^ ^]^ However, several in vitro and in vivo assays are required to validate the effective capacity of the candidate MBGs to trigger ICD.^[^ ^242^ ^]^

## 8 Biomolecular Mechanisms Underlying Anticancer Effects of MBGs

Considering the possibility of cellular and genetic impairment by the biomaterials in patients, the evaluation of the safety of MBGs is a crucial step for developing these materials for biomedical applications. Conversely, biomolecular pathways involved in suppressing/enhancing cancer progression are other essential aspects in developing efficient therapeutics against cancer. Although MBGs have gained immense attention for various clinical applications due to their high biocompatibility, there are limited investigations on the biomolecular mechanisms behind the effects of MBGs on human health. In this respect, the cellular and molecular pathways underlying these biomaterials' anticancer effects should be precisely understood to assist researchers in developing safe and effective manufacturing approaches for MBGs.^[^ ^116^ ^]^

Numerous experimental studies have demonstrated that integrating MBGs with metallic ions (e.g., Ag, Ca, Sr, Cu, Mg, and Zn) imparts further therapeutic qualities, including angiogenesis, anticancer, and anti‐inflammation properties. Indeed, depending on the types of metallic ions and their concentration, each such therapeutic application could be achievable. For instance, copper (Cu) is a vital regulatory factor not only involved in the modulation of cell proliferation, but also in regulation of angiogenesis processes such as stimulation of VEGF, angiogenin, fibronectin, and fibroblast growth factor‐1 and 2 (FGF‐1/2).^[^ ^248^ ^]^ Cu could likewise induce angiogenesis through two main signaling pathways, including hypoxia‐inducible factor‐1 (HIF‐1) that activates the initiation step of the angiogenesis process,^[^ ^249^ ^]^ and mitogen‐activated protein kinase (MAPK) signaling pathway that plays an important role in endothelial cell proliferation, which eventually accelerates wound healing.^[^ ^250^ ^]^ Additionally, Cu in combination with other therapeutic agents could also be used for anticancer purposes. As an example, a research group reported Cu‐doped MBG nanoparticles with photothermal activity for enhancing chemotherapy against bone tumor cells.^[^ ^56^ ^]^ This platform could modulate photothermal effect and enhance the drug loading capacity with a sustained release for a long time simultaneously. The in vitro evaluation showed the lowest cytotoxicity against normal cells and good bioactivity by inducing apatite mineralization formation, offering a remarkable strategy for bone tumor therapy. Mg as another metallic agent is essential to most cellular processes, including energy metabolism, DNA transcription, and protein synthesis, which could induce mTOR activity in tissues, resulting in inhibition of autophagy and promotion of cellular viability.^[^ ^251^ ^]^ Numerous studies have demonstrated that Mg‐doped MBG nanoparticles have reliable bioactivity with high anticancer efficiency, thus representing a promising multifunctional candidate for cancer therapy.^[^ ^252^, ^253^, ^254^ ^]^

Ca^2+^ in MBG nanospheres has been shown to induce transient receptor potential (TRP) channels and calcium‐sensing receptor (CaSR) on tumor cells, which resulted in modulation of the Calpain‐1‐Bcl‐2‐Caspase‐3 signaling pathway to targeted inhibit tumor growth without significant toxicity on normal cells.^[^ ^122^ ^]^ It was also shown that dendritic MBG nanospheres incorporating anticancer drugs could improve the antitumor efficacy and minimize its systemic toxicity. Because the excess intracellular Ca^2+^ causes apoptosis, the effective primary strategy for suppressing tumors, Ca^2+^ addition to MBG could be a promising approach for cancer therapy.^[^ ^255^, ^256^ ^]^

Silver ion (Ag^+^) is another potential anticancer agent that induces cytotoxicity against tumor cells through generating ROS. A recent investigation developed silver oxide (Ag_2_O)‐doped MBG nanoparticles for the controlled release of DOX and evaluated the anticancer activity in human bone tumor cells (MG‐63).^[^ ^118^ ^]^ It was proposed that Ag nanoparticles in combination with DOX could act as potential anticancer agents. Their cytotoxic mechanisms against tumor cells typically trigger oxidative stress, subsequent induction of mitochondrial damage, apoptosis, and DNA damage. Notably, the toxicity evaluation of Ag_2_O‐MBG nanoparticles on normal human fibroblast (NHFB) cells indicated very low toxicity, introducing Ag_2_O‐MBG as a suitable alternative for conventional cancer treatment and angiogenesis inhibitor therapy due to its effective anti‐cancer properties.

**Figure 20:**
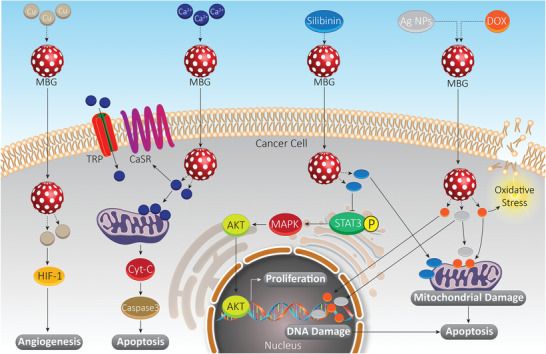
Schematic illustration of some possible effects of ion‐incorporated MBGs for cancer therapy. Apoptosis can be induced by triggering mitochondrial damage and upregulating proapoptotic factors such as caspases. Furthermore, DNA damage and molecular pathways involved in cancer proliferation such as Akt, MAPK, and STAT3 can be modulated. As a major mechanism involved in cancer metastasis, angiogenesis of cancer cells can be regulated by MBGs by affecting HIF signaling pathway. Abbreviations: MBG, mesoporous bioactive glass; HIF, hypoxia‐inducible factor; TRP, transient receptor potential; CaSR, calcium‐sensing receptor; Cyt C, cytochrome C; STAT3, signal transducer and activator of transcription 3; Akt, protein kinase B; MAPK, mitogen‐activated protein kinase; DOX, doxorubicin.

Naturally occurring compounds could also be encapsulated into MBGs to improve their bioavailability, which is an important but challenging issue against their therapeutic impacts. For instance, silibinin, a natural anticancer flavonolignan agent, was encapsulated into MBG nanoparticles and the therapeutic effects against breast tumor cells (MDA‐MB‐231) were studied.^[^ ^34^ ^]^ The results showed that silibinin‐loaded MBGs at two optimal concentrations (9 and 18 µg mL^−1^) could affect the expression of the oncogenic protein (transcription factor) STAT3 (signal transducer and activator of transcription‐3), resulting in disruption of the mitochondria activity and tumor suppression, whereas it exhibited reduced effects on normal cells. Since STAT3 is involved in inducing cancer‐related genes and physically and functionally interacting with other tumor transcription factors, silibinin‐loaded MBG nanoparticles were suggested as a novel and effective agent for safe breast cancer chemotherapy.^[^ ^34^ ^]^ A schematic illustration of some possible therapeutic effects of MBGs combined with ions and chemotherapy agents is shown in **Figure** **20**.

According to the aforementioned in vitro and in vivo studies, the antitumor activity of MBGs against tumor cells has been evaluated. However, just a few biomolecular mechanisms underlying anticancer activity of MBGs have been revealed. To this end, the impact of MBGs on noncoding RNAs, phosphatase, and tensin homolog as an inhibitor of Akt signaling, epithelial‐to‐mesenchymal and so on should be elucidated to show the full capacity of MBGs in targeting and modulating molecular pathways for efficient cancer therapy.

## 9 Paving the Way for Clinical Translation

**Figure 21:**
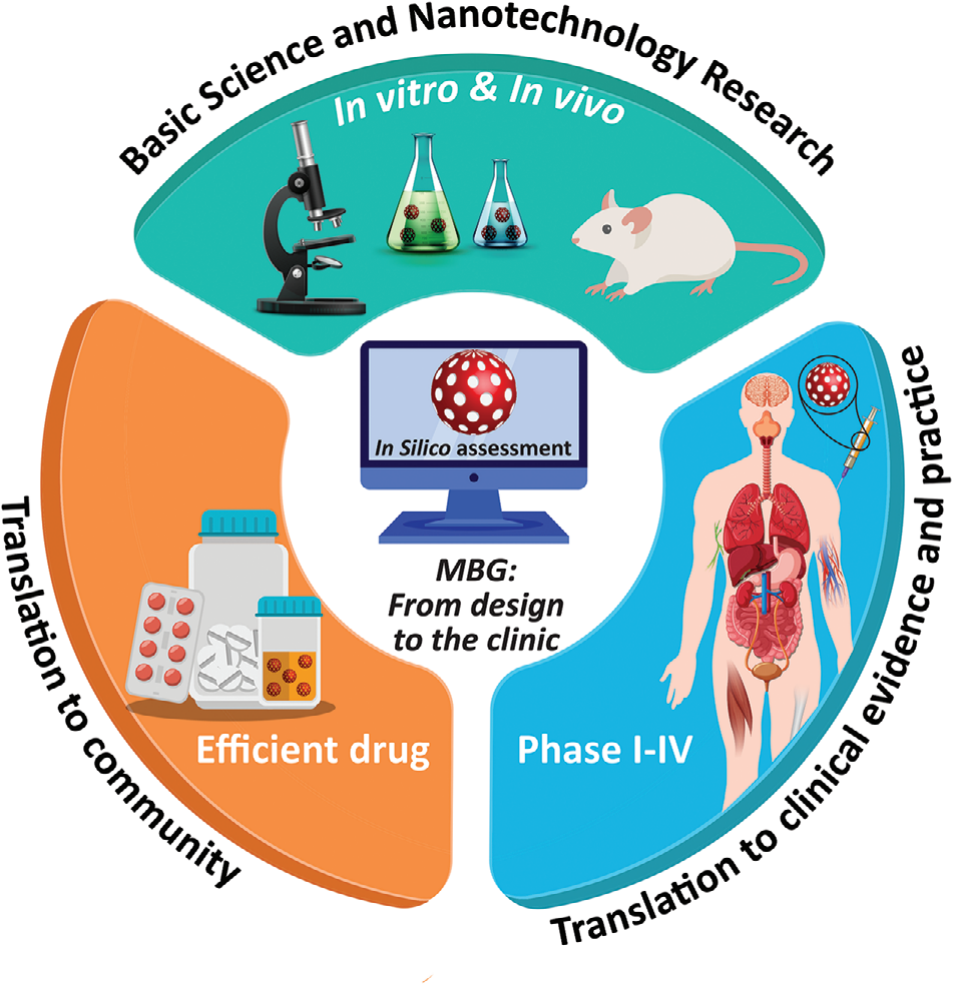
The convergent pathway toward clinical translation of MBGs requires a necessary balance between in vitro, in vivo, and in silico studies.

Translating the knowledge archived in laboratories into clinical trials (bench‐to‐bedside) is an important and challenging issue for applying a safe and efficient cancer treatment.^[^ ^257^ ^]^ Despite the remarkable progress in basic science (preclinical and animal studies), technology, and our understanding of human biology, translating such technologies into practice is not keeping pace with the rising demand. Developing antitumor therapeutics have typically encountered several restrictions in terms of clinical impacts, especially for their side effects against healthy tissues.^[^ ^258^ ^]^ Although numerous in vitro studies have indicated that MBGs combined with chemotherapy agents and ions could suppress tumor cells’ activity with limited toxicity against normal cells, there are limited precise in vivo studies and almost nonexistent clinical research in this field. In light of this, a significant issue that should be carefully investigated in the future is the risk of toxicity in a broad context, including systemic, tissues, and organs affected by MBGs. To promote and accelerate the translation of preclinical investigations into clinical applications, more promising materials are needed to fabricate rational therapeutics that only target tumor sites with minimum side effects. In silico evaluation, state‐of‐the‐art technology to assay approved medicines, decreases the drug's regulatory costs and the time to market for innovation. It is vital to translate human biomedical products into clinical trials and to pave the innovation to the market.^[^ ^259^ ^]^ The combination of in silico assessments and large molecular/drug‐related databases helps select appropriate therapeutics by screening their possible side effects and evaluating their interaction with host cells. To date, several computational strategies and numerous algorithms have been utilized for simulating 3D tumor growth to evaluate large‐scale biological issues.^[^ ^260^ ^]^ However, due to a number of issues, there are limited efficient antitumor drugs.^[^ ^261^ ^]^ To this end, a balance between fundamental discovery, including in vitro and in vivo studies, and applied research may be crucial for developing repurposed drugs, considering the comprehensive chain translating from basic research to clinical evidence and practice to proof of concept.^[^ ^262^ ^]^ This convergent pathway is schematically illustrated in **Figure** **21**. Moreover, to achieve successful translation research from bench‐to‐bedside, rather than focusing on only technology, multidisciplinary collaborations between science and industry is fundamentally required, alter in view of the training of the next generation of researchers.

## 10 Conclusion and Future Perspectives

Compared to conventional BGs, MBGs have opened a new avenue to modern cancer therapy through their extraordinary properties. MBGs are endowed with excellent physical and chemical properties, tailorable morphological, textural, and surface chemistry (functionalization) properties, besides their highly biocompatible nature. Other key features of MBGs are their tunable composition via facile synthesis techniques, cost‐effectiveness, and greater sustainability. MBGs have an amorphous structure and different therapeutic agents with specific capabilities including antibacterial, antiangiogenic, anti‐inflammatory, and anticancer agents can be either embedded or be loaded into the mesopores.^[^ ^71^, ^72^ ^]^ Regarding applications in cancer therapy, the large surface area of MBGs allows the encapsulation of both hydrophilic and hydrophobic anticancer drugs, as well as photodynamic and photothermal agents into their well‐ordered structure with high cargo loading capacity. Such capability makes MBGs excellent candidates to fabricate advanced drug delivery systems with a sustained and/or controlled drug release profile in cancer therapy.^[^ ^263^, ^264^, ^265^ ^]^ In addition, several ions with anticancer and regenerative properties can be integrated into the framework of MBGs with excellent contribution in tissue engineering^[^ ^25^ ^]^ and cancer therapy.^[^ ^23^ ^]^ Moreover, MBGs enable the simultaneous delivery of therapeutic ions and drugs to obtain synergistic outcomes.^[^ ^28^ ^]^ Although, monotherapy using MBGs is generally used to ablating tumors, complete tumor eradication, the killing of deep‐seated tumors is still a significant challenge in this arena.^[^ ^266^ ^]^ The design and implementation of combination therapies using chemotherapy, photodynamic therapy, photothermal therapy, immunotherapy, radiotherapy, and gene therapy to achieve a complementary multitherapeutic efficacy are of especial importance to overcome the hard to ignore limitation.^[^ ^267^ ^]^ In addition, the vital issues of large‐scale production, reproducibility, precise physiochemical property and surface modification, desirable biological effect as well as underlying therapeutic mechanism of MBG‐based formulations should be further resolved in future investigations. Moreover, the combination of regenerative biomaterials based on MBGs with concurrent therapeutic and regeneration approaches for versatile biomedical applications is still in its infancy. By using MBG‐derived multifunctional biomaterials not only simultaneous bone tissue regeneration/bone cancer treatment can be achieved, but also MBGs have the potential to be beneficial in contact with other tissues and organs including skin‐cancer therapy/skin tissue regeneration, skin infection therapy/skin tissue repair, osteoarticular tuberculosis therapy/bone tissue regeneration, and so on.

As foreign materials, MBGs might be potentially immunogenic and trigger harmful immune responses. In this scenario, a deeper understanding of the impact of MBGs on the host's immune system would be fundamental to guide the design of safe therapeutic products based on these nanomaterials. Recently, in vitro studies have assessed the immunotoxicity of MBGs and confirmed their biocompatibility.^[^ ^35^, ^229^, ^238^ ^]^ Also, inducing macrophage polarization toward the anti‐inflammatory M2 phenotype, MBGs might be considered as a suitable platform for nanomedicine applications without triggering chronic inflammation. Nevertheless, there are still some unsolved biological aspects of these materials for reliable cancer therapy, including the optimal dosage of therapeutic molecules (e.g., ions, drugs) for incorporating into MBGs, the long‐term side effects of MBGs, and the in vivo fate of MBGs. It is crucial to first study the potential of these materials as safe and potent drug delivery structures through performing well‐organized preclinical and clinical studies. The ideas reported in this article explore fundamental issues of BGs, specifically MBGs, in combination with nanotechnology to offer an overview of the potential for these carriers for subsequent clinical trials. In this regard, multifunctional MBGs have shown efficient drug delivery capability with high bioactivity, making them good candidates for clinical applications. The authors anticipate that the combination of MBGs and targeting moieties will offer increased safety for more precise treatment of cancer in the near future.

## Conflict of Interest

The authors declare no conflict of interest.
